# Joint estimation of source dynamics and interactions from MEG data

**DOI:** 10.1162/netn_a_00453

**Published:** 2025-07-17

**Authors:** Narayan Puthanmadam Subramaniyam, Filip Tronarp, Simo Särkkä, Lauri Parkkonen

**Affiliations:** Department of Neuroscience and Biomedical Engineering, Aalto University, Espoo, Finland; Department of Electrical Engineering and Automation, Aalto University, Espoo, Finland

**Keywords:** MEG, Bayesian filtering, Functional connectivity, Source localization

## Abstract

Current techniques to estimate directed functional connectivity from magnetoencephalography (MEG) signals involve two sequential steps: (a) estimation of the sources and their amplitude time series from the MEG data and (b) estimation of directed interactions between the source time series. However, such a sequential approach is not optimal as it leads to spurious connectivity due to spatial leakage. Here, we present an algorithm to jointly estimate the source and connectivity parameters using Bayesian filtering. We refer to this new algorithm as JEDI-MEG (Joint Estimation of source Dynamics and Interactions from MEG data). By formulating a state-space model for the locations and amplitudes of a given number of sources, we show that estimation of their connections can be reduced to a system identification problem. Using simulated MEG data, we show that the joint approach provides a more accurate reconstruction of connectivity parameters than the conventional two-step approach. Using real MEG responses to visually presented faces in 16 subjects, we also demonstrate that our method gives source and connectivity estimates that are both physiologically plausible and largely consistent across subjects. In conclusion, the proposed joint estimation approach outperforms the traditional two-step approach in determining functional connectivity in MEG data.

## Author Summary

Functional connectivity is currently estimated from electromagnetic brain signals such as magnetoencephalography (MEG) in two consecutive steps: First, the inverse problem is solved to estimate the locations and temporal dynamics of brain sources. Second, connectivity metrics are computed between these estimated sources. This approach suffers from the limitation that the information provided by the connectivity structure is not exploited in the estimation of source activity and vice versa. Here, we present a novel algorithm, utilizing Bayesian filtering, to jointly estimate the source and connectivity parameters to overcome this limitation. Compared with state-of-the-art two-step approaches, our method provides a more accurate reconstruction of the connectivity parameters, which we demonstrate using a standard connectivity benchmark simulation and an electrocorticography-based simulation of MEG data. We also applied our method to real MEG responses (open-access dataset) to visually presented faces in 16 subjects, and the results show that our approach provides source and connectivity estimates that are both physiologically plausible and largely consistent across subjects. Overall, this work contributes to methodological advances in estimating functional connectivity from MEG data.

## INTRODUCTION

The two fundamental and contrasting principles of cortical organization are functional segregation and integration, which span multiple spatiotemporal scales. While functional segregation refers to the existence of functionally specialized brain areas that are anatomically separated, functional integration refers to the coordinated interaction between these specialized brain regions, which is essential for various cognitive and perceptual tasks (Bastos & Schoffelen, 2016). The investigation of functional relationships between brain regions has been a major task in neuroscience since the beginning of electroencephalography (EEG). It has been hypothesized that neuronal oscillations and their interregional synchronization is essential to normal brain function. Being able to measure and characterize brain signals reflecting functional integration shall help us understand how connections between brain regions mediate information and how such connections change, for example, due to learning or a neurodegenerative disease.

Functional integration is usually described in terms of functional connectivity (Friston, 2011), which is defined as a statistical interdependency of activation dynamics in distinct brain regions. Activation dynamics can be measured indirectly with functional magnetic resonance imaging (fMRI) as fluctuations of the blood-oxygen-level-dependent signal or directly with magnetoencephalography (MEG) or EEG as changes in the electromagnetic signals emitted by electrically active neurons. Owing to their millisecond-range temporal resolution, EEG and MEG are ideal methods to monitor the dynamic neural signals.

However, due to their limited spatial resolution, estimating functional connectivity directly from MEG or EEG is not a straightforward task, and it typically comprises two sequential steps. In the first step, the ill-posed inverse problem of estimating the source activities from MEG and/or EEG signals is solved, for instance, using minimum-norm estimation (MNE; Hämäläinen & Ilmoniemi, 1994) or beamforming (Sekihara & Scholz, 1996; Van Veen, van Drongelen, Yuchtman, & Suzuki, 1997). In the second step, regions of interest (ROIs) based on anatomical atlases or separate functional localizer measurements are defined, and the estimated activities of all sources in a given ROI are collapsed to obtain representative activity dynamics for each ROI. Functional connectivity can then be estimated between a subset or all the ROIs using a variety of methods. While measures such as coherence, phase-locking value, and phase-lag index yield a symmetric connectivity matrix, approaches based on multivariate autoregressive (MVAR) models can result in an asymmetric connectivity matrix, which directed connectivity measures such as partial directed coherence (PDC) and directed transfer function exploit. With these directed measures, the sources (or ROIs) are typically assumed to be few and their locations known.

Since the estimation of source activities and the connectivity between them occur in two distinct steps, the connectivity estimation method is not informed about the assumptions and limitations the source-estimation algorithm makes, for example, independence across the sources, which eventually leads to biased connectivity estimates. In particular, when distributed source estimates, such as MNE, are employed, two-step approaches often lead to spurious connections between sources.

To include additional information in source estimation, linear MVAR models with spatially local interactions and self-interactions have been proposed (Fukushima et al., 2012; Galka, Yamashita, Ozaki, Biscay, & Valdés-Sosa, 2004; Lamus, Hämäläinen, Temereanca, Brown, & Purdon, 2012). However, such approaches ignore long-range interactions across brain regions, which are the hallmark of functional integration.

Joint estimation of source activities and connectivity has been popularly addressed by a confirmatory approach known as dynamic causal modeling (DCM; Friston, Harrison, & Penny, 2003). In DCM, sources are prespecified, and the activity in each ROI is modeled through a set of nonlinear differential equations. Then, the estimated connectivity solutions for plausible generative models are compared using Bayesian model selection, and an appropriate model is chosen.

Over the last few years, a data-driven, exploratory approach has been used for addressing the joint estimation problem using MVAR models (Cheung, Riedner, Tononi, & Van Veen, 2010; Subramaniyam, Tronarp, Särkkä, & Parkkonen, 2017). In this approach, the expectation–maximization (EM) approach with a Kalman smoother in a state-space formulation is used to obtain a maximum a posteriori estimate of the source signals and a maximum likelihood (ML) estimate of the MVAR coefficients, which describe the interaction. However, these approaches still require the ROIs or source locations to be predefined. Recently, Fukushima, Yamashita, Knösche, and Sato (2015) proposed a joint estimation of whole-brain connectivity from MEG signals without requiring ROIs to be predefined but relying on structural connectivity information, for example, from diffusion tensor imaging. However, such information may not be available, and functional connectivity patterns typically only loosely follow structural connectivity.

In this work, we propose a framework for the joint estimation of source locations, amplitudes, and their directed functional connections from MEG data, which we refer to as JEDI-MEG (Joint Estimation of source Dynamics and Interactions from MEG) data. We formulate a state-space model (SSM) for the evolution of source locations and source amplitudes. The transition model for source locations is a first-order random walk, while for source amplitudes, it is a *P*th order MVAR model, with the coefficients representing the interaction between the sources.

Estimation of the MVAR coefficient matrix is then reduced to a system identification problem in a nonlinear state space with a tractable linear substructure, which can be solved efficiently using the stochastic approximation expectation–maximization (SAEM) approach combined with Rao–Blackwellization (RB; Svensson, Schön, & Lindsten, 2014).

Finally, the system identification problem of MVAR matrix estimation is solved with an ML approach. Once the MVAR matrix is estimated, directed functional connectivity estimates can then be obtained using methods such as generalized PDC (gPDC; Baccalá, Sameshima, & Takahashi, 2007), which provides a frequency-domain representation of the MVAR model.

To evaluate the performance of JEDI-MEG against two-step approaches, we used MVAR-based as well as electrocorticography (ECoG)-based simulations. We also applied our methods to real MEG data, where the subjects (*N* = 16) were shown human faces.

## MATERIALS AND METHODS

### Notation and Assumptions

We represent a multivariate Gaussian distribution of **x** with mean ***μ*** and covariance **Σ** as 𝓝(**x**|***μ***, **Σ**). The predicted and filtered mean of the Kalman filter are denoted as ***μ***_*t*|1:*t*−1_ and ***μ***_*t*|1:*t*_, respectively, whereas ***μ***_*t*|1:*T*_ refers to the mean of the Kalman smoother. In a similar vein, we define **P**_*t*|1:*t*−1_, **P**_*t*|1:*t*_, and **P**_*t*|1:*T*_ for the Kalman filter and smoother covariances.

We denote the number of MEG time samples as *T* and number of trials as *J*. The *M*-channel MEG measurement of the *j*th trial at time *t* is denoted as $y_{t}^{j}$ ∈ ℝ^*M*×1^. We assume that only a reasonable number of sources (1 < *N*_*s*_ ≤ 5) are active and they can be modeled as dipoles oriented normal to the local cortical surface. We further assume that the source locations remain constant across the trials. We denote the location of *N*_*s*_ sources at time *t* as **r**_*t*_ ∈ ℝ^3*N*_*s*_×1^. The amplitudes of *N*_*s*_ sources at time *t* and trial *j* are denoted as $q_{t}^{j}$ ∈ ℝ^*N*_*s*_×1^. We define **x**_1:*t*_ ≜ {**r**_1:*t*_, **q**_1:*t*_}. Note that **q**_1:*t*_ ≜ {$q_{1}^{1}\ldots q_{1}^{J}\ldots q_{t}^{1}\ldots q_{t}^{J}$}. In a similar vein, we define **y**_1:*t*_. Furthermore, we assume that the number sources is known a priori, and all the noise in the model has a Gaussian distribution with zero mean.

### Model for Source and Connectivity Dynamics

We formulate an SSM of the form

$$\begin{array}{l} r_{t}=m_{t-1}+\xi_{t}\\ q_{t}^{j}=\sum_{p=1}^{P}A_{p}q_{t-p}^{j}+v_{t}^{j}\\ y_{t}^{j}=G\left(r_{t}\right)q_{t}^{j}+e_{t}^{j}. \end{array}$$ (1)

The matrix **A***_p_* consists of the MVAR coefficients representing the correlation between the sources at time-delay *t* − *p*, across all the trials. **G**(**r**_*t*_) ∈ ℝ^*M*×*N*_*s*_^ is the lead-field matrix describing the contribution of *N*_*s*_ dipoles of unit strength at location **r***_t_* to *M* MEG sensors. The process noise for the source amplitudes is given by $v_{t}^{j}∼𝓝\left(v_{t}^{j}|0,V\right)$. The measurement noise is denoted by $e_{t}^{j}∼𝓝\left(e_{t}^{j}|0,E\right)$.

The source locations do not depend on individual trials but are rather estimated from the average across the trials. We incorporate an artificial evolution for source locations in our SSM since we use a particle filter to estimate them. However, as it is commonly assumed in several dipole-localization approaches, the true locations of the sources are fixed in time and only their amplitudes vary. To estimate the fixed source locations using a particle filter, we adopt a kernel smoothing approach via shrinkage as described in Liu and West (2001). The kernel location is given by **m**_*t*−1_ and is specified using a shrinkage rule. The artificial evolution noise for source locations is given as ***ξ***_*t*_ ∼ 𝓝(***ξ***_*t*_|**0**, *h*^2^**Ξ**), where *h* is the smoothing parameter. The kernel smoothing approach is further explained in Supporting Information Section S2. To facilitate the application of Bayesian filtering algorithms, a *P*th order MVAR model has to be reformulated into a first-order MVAR model as follows:

$$\begin{array}{l} r_{t}=m_{t-1}+\xi_{t}\\ {\tilde{q}}_{t}^{j}=\tilde{A}{\tilde{q}}_{t-1}^{j}+{\tilde{v}}_{t}^{j}\\ y_{t}^{j}=G\left(r_{t}\right)q_{t}^{j}+e_{t}^{j} \end{array}$$ (2)

where $\tilde{A}$, $\tilde{V}$, and $\tilde{q}$ are described in Supporting Information Section S1.

### JEDI-MEG

**Figure 1.**
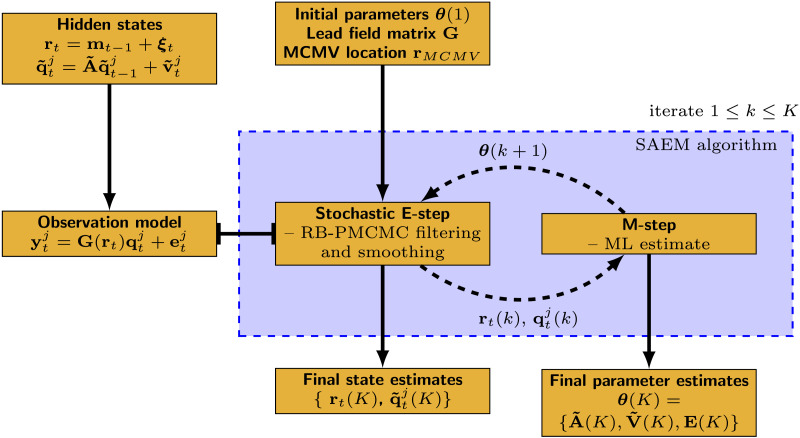
Schematic illustration of the JEDI-MEG method.

The JEDI-MEG method uses a variant of the EM algorithm known as stochastic approximation EM (SAEM) algorithm; it employs a particle Markov chain Monte-Carlo (PMCMC) method to estimate source locations and a Kalman filter to estimate the source amplitudes within the expectation-step (stochastic E-step) of the SAEM algorithm. The connectivity matrix and other covariance matrices are obtained via ML estimation in the maximization-step (M-step). We describe the JEDI-MEG method in detail below; see also Figure 1 for a schematic illustration.

Given a latent variable **x**_1:*T*_ and measurement **y**_1:*T*_, an EM algorithm employs an iterative scheme where the auxiliary quantity

$$𝒬\left(\theta ,\theta^{\prime }\right)=\int \log p_{\theta }\left(x_{1:T},y_{1:T}\right)p_{\theta^{\prime }}\left(x_{1:T}|y_{1:T}\right){dx}_{1:T}$$ (3)

related to the lower bound of the marginal log-likelihood is maximized as opposed to the direct maximization of the marginal log-likelihood, which is not always possible. Starting from an initial guess ***θ***[0] ∈ **Θ**, an EM algorithm iterates between the two steps until convergence or the maximum number of iterations is reached:

E-step: Compute 𝒬(***θ***, ***θ***[*k* − 1])
M-step: ***θ***[*k*] ← arg max_Θ_ 𝒬(***θ***, ***θ***[*k* − 1]).

At every *k*th iteration, we get the full posterior or the joint smoothing distribution, *p*_*θ*[*k*−1]_(**x**_1:*T*_|**y**_1:*T*_), which solves the state inference problem related to the estimation of source locations and strength. In the case of nonlinear SSMs, the E-step does not have a closed-form solution and needs to be approximated using a stochastic version of the EM algorithm, where a Monte Carlo approach can be used to approximate the the integral in Equation 3 (Kuhn & Lavielle, 2004). However, such methods are inefficient, since at each new iteration the values simulated during the previous iteration are completely discarded.

Deylon and colleagues (Delyon, Lavielle, & Moulines, 1999) proposed an alternative scheme, known as the SAEM algorithm, where the simulated variables are gradually discarded with each iteration using a forgetting factor *ζ*_*k*_. In SAEM, the E-step is replaced by a stochastic E-step:

$${\hat{𝒬}}_{k}\left(\theta \right)=\left(1-\zeta_{k}\right){\hat{𝒬}}_{k-1}\left(\theta \right)+\zeta_{k}\log p_{\theta }\left(x_{1:T}\left[k\right],y_{1:T}\right)$$ (4)

where **x**_1:*T*_[*k*] ∼ *p*_*θ*[*k*−1]_(**x**_1:*T*_|**y**_1:*T*_) and {*ζ*_*k*_}_*k*≥1_ is a decreasing sequence satisfying the constraints ∑_*k*_*ζ*_*k*_ = ∞ and ∑_*k*_$\zeta_{k}^{2}$ < ∞ (Lindsten, 2013).

Given the MEG observations **y**_1:*T*_ and the number of sources *N*_*s*_, we estimate the state **x**_1:*T*_ comprising the locations **r**_1:*T*_ and amplitudes $\tilde{q}$_1:*T*_ of the sources using RB procedure within the stochastic E-step, and the parameters ***θ*** = {$\tilde{A}$, $\tilde{V}$, **E**} comprising of the autoregressive (AR) coefficient matrix $\tilde{A}$, process-noise covariance matrix for the source amplitudes $\tilde{V}$, and measurement-noise covariance matrix **E** using a maximum-likelihood (ML) approach within the M-step. We briefly explain these approaches in the RB, Estimating Source Locations, Estimating Source Amplitudes, and ML Estimation of the Parameters sections.

#### RB.

Making use of conditional probabilities, we can express the target distribution of interest at SAEM iteration *k* as

$$\begin{array}{l} p_{\theta \left[k\right]}\left(x_{1:T}|y_{1:T}\right)=p_{\theta \left[k\right]}\left(r_{1:T},{\tilde{q}}_{1:T}|y_{1:T}\right)\\ \;=p_{\theta \left[k\right]}\left(r_{1:T}|y_{1:T}\right)p_{\theta \left[k\right]}\left({\tilde{q}}_{1:T}|r_{1:T},y_{1:T}\right). \end{array}$$ (5)

Since the distribution *p*_*θ*[*k*]_($\tilde{q}$_1:*T*_|**r**_1:*T*_, **y**_1:*T*_) is Gaussian, the inference of the conditionally linear states $\tilde{q}$_1:*T*_ can be done in closed form using a Kalman filter and smoother (Särkkä, 2013), thus requiring the Monte Carlo-based simulation method only to approximate *p*_*θ*[*k*]_(**r**_1:*T*_|**y**_1:*T*_). Such marginalization, known as RB (Casella & Robert, 1996; Särkkä, 2013), reduces the asymptotic variance of the estimator as well as the computational cost.

As mentioned previously, we will use the trial-averaged MEG data

$${\bar{y}}_{t}=\frac{1}{J}\sum_{j=1}^{J}y_{t}^{j}$$ (6)

to estimate the source locations. We employ the PMCMC method to approximate the posterior distribution *p*_*θ*[*k*]_(**r**_1:*T*_|$\bar{y}$_1:*T*_) of the source locations, and a Kalman smoother to estimate the posterior *p*_*θ*[*k*]_($\tilde{q}$_1:*T*_|**r**_1:*T*_, **y**_1:*T*_) of the amplitudes, which is referred to as the RB-PMCMC approach (Lindsten, 2013).

#### Estimating source locations.

Instead of sampling directly from the posterior distribution *p*_*θ*[*k*]_(**r**_1:*T*_|$\bar{y}$_1:*T*_), it is sufficient to generate samples using a Markov kernel, which is assumed to be ergodic with the stationary distribution *p*_*θ*[*k*]_(**r**_1:*T*_|$\bar{y}$_1:*T*_). Let Π_*θ*_(·) be such a kernel. We then have

$$r_{1:T}\left[k\right]∼\Pi_{\theta \left[k-1\right]}\left(\cdot |r_{1:T}\left[k-1\right]\right).$$ (7)

By simulating a Markov chain with the kernel Π_*θ*_(·), the marginal distribution of the chain will approach the posterior distribution *p*_*θ*[*k*]_(**r**_1:*T*_|$\bar{y}$_1:*T*_) for a sufficiently large *k* (Svensson et al., 2014). PMCMC approaches can be used to construct an efficient and high-dimensional kernel Π_*θ*_(·). In PMCMC, a sequential Monte Carlo sampler, such as a particle filter, is used as the Markov kernel. As proposed by Lindsten (2013), we use a variant of the particle filter known as conditional particle filter with ancestor sampling (CPF-AS) as the kernel. Using CPF-AS, we approximate the posterior distribution at iteration *k* *p*_*θ*[*k*]_(**r**_1:*t*_|$\bar{y}$_1:*t*_) as (Lindsten, Jordan, & Schön, 2014)

$${\hat{p}}_{\theta \left[k\right]}\left({\mathrm{dr}}_{1:t}|{\bar{y}}_{1:t}\right)=\sum_{i=1}^{N_{p}}w_{t}^{i}\delta_{r_{1:t}^{i}}\left({\mathrm{dr}}_{1:t}\right)$$ (8)

where *δ*(·) is an impulse function and ${\left\{r_{1:t},w_{t}^{i}\right\}}_{i=1}^{N_{p}}$ is a weighted particle system with *N*_*p*_ particles such that ∑_*i*_ $w_{t}^{i}$ = 1. The CPF-AS approach is similar to particle filtering except that the trajectory of the *N*_*p*_th particle is set deterministically. In this work, instead of sampling from a distribution (Lindsten, 2013), we use the location estimates provided by the MCMV algorithm to initialize the trajectory of *N*_*p*_th particle, that is, $r_{1:T}^{N_{p}}$ = **r**_MCMV_. Details of the CPF-AS method are given in Supporting Information Section S2.

#### Estimating source amplitudes.

Conditioned on the source locations, $r_{t}^{i}$ for *i* = 1, 2, …, *N*_*p*_, the predictive distribution of ${\tilde{q}}_{t}^{i,j}$ is given as

$$p\left({\tilde{q}}_{t}^{i,j}|y_{1:t-1}^{j},r_{1:t}^{i}\right)=𝓝\left({\tilde{q}}_{t}^{i,j}|\mu_{t|1:t-1}^{i,j},P_{t|1:t-1}^{i,j}\right)$$ (9)

and the filtering distribution of ${\tilde{q}}_{t}^{i,j}$ is given as

$$p\left({\tilde{q}}_{t}^{i,j}|y_{1:t}^{j},r_{1:t}^{i}\right)=𝓝\left({\tilde{q}}_{t}^{i,j}|\mu_{t-1|1:t-1}^{i,j},P_{t-1|1:t-1}^{i,j}\right).$$ (10)

We use a Rauch–Tung–Striebel (RTS) smoother to obtain the marginal smoothing distribution of ${\tilde{q}}_{t}^{i}$, which is given as

$$p\left({\tilde{q}}_{t}^{i,j}|r_{1:T}^{i},y_{1:T}^{j}\right)=𝓝\left({\tilde{q}}_{t}^{i,j}|\mu_{t|1:T}^{i,j},P_{t|1:T}^{i,j}\right).$$ (11)

From the RTS smoother, we also obtain the one-lag Kalman smoother covariance, $P_{t,t-1|1:T}^{i,j}≜\text{cov}\left(\mu_{t-1|T}^{i,j},\mu_{t|T}^{i,j}\right)$. The explicit expressions of the mean and covariance of the Kalman filter and smoother are given in Supporting Information Section S3.

At iteration *k* of the SAEM algorithm, we update $\hat{𝒬}$_*k*_(***θ***) in the stochastic E-step given in Equation 4 by making use of all the particles given by CPF-AS instead of just using **r**_1:*T*_[*k* + 1] (Lindsten, 2013). That is,

$${\hat{𝒬}}_{k}\left(\theta \right)=\left(1-\zeta_{k}\right){\hat{𝒬}}_{k-1}\left(\theta \right)+\zeta_{k}\sum_{i=1}^{N_{p}}w_{T}^{i}{𝔼}_{\theta \left[k-1\right]}\left[\log p_{\theta \left[k-1\right]}\left(y_{1:T},{\tilde{q}}_{1:T},r_{1:T}^{i}\right)\right]$$ (12)

where the 𝔼_*θ*[*k*−1]_[·] is with respect to the linear state $\tilde{q}$_1:*T*_ obtained by the Kalman smoother. For notational convenience, we refer to $r_{1:T}^{i}$[*k*] and $w_{T}^{i}$[*k*] as simply $r_{1:T}^{i}$ and $w_{T}^{i}$.

#### ML estimation of the parameters.

Connectivity and noise covariance parameter estimates are obtained in the maximization step of the SAEM method, where our aim is to maximize $\hat{𝒬}$_*k*_(***θ***) of Equation 12, with respect to ***θ*** = {$\tilde{A}$, $\tilde{V}$, **E**}.

The complete log-likelihood in Equation 12 can be factorized as

$$\begin{array}{l} \log p_{\theta }\left({\tilde{q}}_{1:T},r_{1:T}^{i},y_{1:T}\right)=\sum_{t=1}^{T}\log p\left(r_{t}^{i}|r_{t-1}^{i}\right)\\ \;+\sum_{t=1}^{T}\sum_{j=1}^{J}\log p_{\theta }\left({\tilde{q}}_{t}^{j}|{\tilde{q}}_{t-1}^{j}\right)\\ \;+\sum_{t=1}^{T}\sum_{j=1}^{J}\log p_{\theta }\left(y_{t}^{j}|r_{t},{\tilde{q}}_{t}^{j}\right). \end{array}$$ (13)

Only the second and third term in the above expression depend on ***θ***, and thus taking the expectation of only this part of the joint log-likelihood, we have (Särkkä, 2013):

$$\begin{array}{l} {𝔼}_{\theta \left[k\right]}\left[\log p_{\theta }\left({\tilde{q}}_{1:T},r_{1:T}^{i},y_{1:T}\right)\right]\\ ={𝔼}_{\theta \left[k\right]}\left[\sum_{t=1}^{T}\sum_{j=1}^{J}\log p_{\theta }\left({\tilde{q}}_{t}^{j}|{\tilde{q}}_{t-1}^{j}\right)+\sum_{t=1}^{T}\sum_{j=1}^{J}\log p_{\theta }\left(y_{t}^{j}|r_{t},{\tilde{q}}_{t}^{j}\right)\right]\\ =-\frac{JT}{2}\log\left|2\pi \tilde{V}\right|-\frac{JT}{2}\log\left|2\pi E\right|-\frac{1}{2}tr\left\{{\tilde{V}}^{-1}\left[\Phi -\Psi {\tilde{A}}^{\text{T}}-\tilde{A}\Psi^{\text{T}}+\tilde{A}\Sigma {\tilde{A}}^{\text{T}}\right]\right\}\\ \;-\frac{1}{2}tr\left\{E^{-1}\left[Z-ϒ{\bar{G}}^{\text{T}}-\bar{G}{ϒ}^{\text{T}}+\bar{G}\Phi {\bar{G}}^{\text{T}}\right]\right\} \end{array}$$ (14)

where $\bar{G}=\frac{1}{N_{p}T}\sum_{t=1}^{T}\sum_{i=1}^{N_{p}}G\left(r_{t}^{i}\right)$, **E** = $\sigma_{m}^{2}$**I**, and

$$\begin{array}{l} \Phi =\sum_{t=1}^{T}\sum_{j=1}^{J}\sum_{i=1}^{N_{p}}w_{i,T}\left(P_{t|1:T}^{i,j}+\mu_{t|1:T}^{i,j}{\left(\mu_{t|1:T}^{i,j}\right)}^{\text{T}}\right)\\ \Psi =\sum_{t=1}^{T}\sum_{j=1}^{J}\sum_{i=1}^{N_{p}}w_{i,T}\left(P_{t,t-1|1:T}^{i,j}+\mu_{t|1:T}^{i,j}{\left(\mu_{t-1|1:T}^{i,j}\right)}^{\text{T}}\right)\\ \Sigma =\sum_{t=1}^{T}\sum_{j=1}^{J}\sum_{i=1}^{N_{p}}w_{i,T}\left(P_{t-1|1:T}^{i,j}+\mu_{t-1|1:T}^{i,j}{\left(\mu_{t-1|1:T}^{i,j}\right)}^{\text{T}}\right)\\ Z=\sum_{t=1}^{T}\sum_{j=1}^{J}y_{t}^{j}{\left(y_{t}^{j}\right)}^{\text{T}}\\ ϒ=\sum_{t=1}^{T}\sum_{j=1}^{J}y_{t}^{j}\sum_{i=1}^{N_{p}}w_{i,T}{\left(\mu_{t|1:T}^{i,j}\right)}^{\text{T}}. \end{array}$$ (15)

Collecting the sufficient statistics computed above using the Kalman smoother into a matrix

$$S=\left[\begin{array}{cc} \Phi & \Psi \\ \Psi^{\text{T}} & \Sigma \\ Z & ϒ\\ {ϒ}^{\text{T}} & \Phi \end{array}\right],$$ (16)

the computation of the stochastic E-step given in Equation 12 reduces to recursively updating

$${𝕊}_{k}=\left(1-\zeta_{k}\right){𝕊}_{k-1}+\zeta_{k}S_{k}.$$ (17)

At iteration *k*, maximizing $\hat{𝒬}$_*k*_(***θ***) w.r.t $\tilde{A}$, $\tilde{V}$, and **E** (Särkkä, 2013) leads to the following update equations (derivation given in Supporting Information Section S4):

$$\begin{array}{l} {\tilde{A}}_{k}=\Psi \Sigma^{-1}\\ {\tilde{V}}_{k}=\frac{1}{JT}\left(\Phi -\Psi \Sigma^{-1}\Psi^{\text{T}}\right)\\ \sigma_{m,k}^{2}=tr\left\{\frac{1}{MJT}\left(Z-ϒ{\bar{G}}^{\text{T}}-\bar{G}{ϒ}^{\text{T}}+\bar{G}\Phi {\bar{G}}^{\text{T}}\right)\right\}. \end{array}$$ (18)

We also account for the brain noise in our SSM by modifying the measurement equation to

$$y_{t}^{j}=G\left(r_{t}\right)q_{t}^{j,s}+{Gq}_{t}^{j,b}+e_{t}^{j}.$$ (19)

The noise covariance is now given as *σ*^2^ = $\sigma_{m}^{2}$**I** + $\sigma_{b}^{2}$**GG**^T^, where the additional term $\sigma_{b}^{2}$**GG**^T^ represents brain noise projected to the MEG sensors. We assume brain noise to be white, that is, $q_{t}^{j,b}∼𝓝\left(0,\sigma_{b}^{2}I\right)$. In this scenario, the closed-form update for $\sigma_{m}^{2}$ and $\sigma_{b}^{2}$ no longer exists, and we use a gradient-descent algorithm with backtracking to update $\sigma_{m}^{2}$ and $\sigma_{b}^{2}$. The implementation of the SAEM-based algorithm and the RB-PMCMC method within it is described in Supporting Information Section S6.

### Reference Source Estimation With Beamformers and MVAR Fitting

For comparing our method with the conventional two-step approaches, we performed source estimation also with linearly constrained minimum variance (LCMV) and multiple constrained minimum variance (MCMV) beamformers. Briefly, in the case of LCMV, the neural activity index (NAI; Van Veen et al., 1997) was computed for each point in the source space, while for the MCMV beamformer, as proposed by Moiseev and colleagues (Moiseev, Gaspar, Schneider, & Herdman, 2011), multi-activity index (MAI) was computed using an iterative procedure, where at each step the already-localized sources from previous steps remain fixed and only the next source is searched by scanning the whole source space. Once the spatial filters corresponding to the sources were estimated, we computed the source activity time series,

$$s_{t}^{j}=W^{\text{T}}y_{t}^{j},$$ (20)

where **W** ∈ ℝ^*M*×*N*_*s*_^ contains the spatial filter weights estimated by the beamformer (LCMV or MCMV), which is given by,

$$W={\left(G_{\text{sub}}^{\text{T}}C^{-1}G_{\text{sub}}\right)}^{-1}G_{\text{sub}}^{\text{T}}C^{-1}.$$ (21)

Here, **G**_sub_ ∈ ℝ^*M*×*N*_*s*_^ is a subset of lead-field matrix **G** corresponding to *N*_*s*_ source locations with the largest NAI (for LCMV) or MAI (for MCMV) values and **C** is the data covariance matrix. The pipeline for connectivity estimation using beamformers is shown in Supporting Information Section S5.

In case of both LCMV and MCMV beamformers, we regularized the covariance matrix by replacing *C* with (**C** + *λ***I**), where *λ* is the regularization parameter and **C** is the data covariance matrix. We set *λ* as (Jaiswal et al., 2020; Tikhonov, 1963)

$$\lambda =0.05\cdot \frac{\text{Trace}\left(C\right)}{M},$$ (22)

where *M* is the number of channels.

Once the source time-series were estimated using LCMV or MCMV methods, an MVAR model was fitted to the LCMV or MCMV estimated activity using using ARFIT algorithm (Neumaier & Schneider, 2001).

### Model Order Selection

To estimate the model order (for MVAR fitting and JEDI-MEG method), we used Bayesian information criteria (BIC) and varied *P* from 1 to 20. In BIC, the aim is to find the *P* at which the following function attains its minimum:

$${\text{BIC}}_{P}=-2LL+K_{P}\log\left(JT\right),$$ (23)

where *LL* is the log-likelihood.

In case of the JEDI-MEG approach, it is given as

$$LL=-\frac{JT}{2}\log\left|2\pi \tilde{V}\right|-\frac{1}{2}tr\left\{{\tilde{V}}^{-1}\left[\Phi -\Psi {\tilde{A}}^{T}-\tilde{A}\Psi^{T}+\tilde{A}\Sigma {\tilde{A}}^{T}\right]\right\}$$ (24)

and *K*_*P*_ = $N_{s}^{2}$*P* + *N*_*s*_ + 1 is the number of free parameters. Here, $N_{s}^{2}$*P* refers to the number of free parameters in $\tilde{A}$, *N*_*s*_ refers to the number of free parameters in $\tilde{V}$ (i.e., only the diagonal entries of **V**), and free parameter for the brain and measurement noise variance $\sigma_{b}^{2}$ and $\sigma_{m}^{2}$, respectively.

### Simulations Based on MVAR Models

**Figure 2.**
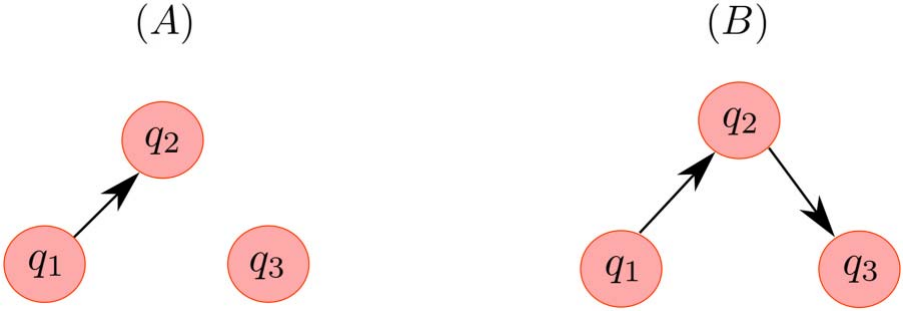
MVAR simulations. (A) Unidirectional interaction between the two sources *q*_1_ and *q*_2_ with *q*_3_ as a noninteracting source (Type-I). (B) Unidirectional interactions between the three sources (Type-II).

We designed our first simulations based on an MVAR model described in Equation 25. To simulate MEG signals (magnetometers), we used a forward model constructed from one of the subjects from the data made available by Wakeman and Henson (2015). To account for modeling errors, where we used a single-layer boundary element method (BEM) model from one subject (see Head Modeling section) with icosahedron subdivision to 5,120 nodes (using “ico4” in MNE-Python; Gramfort et al., 2014) for data generation and for estimation we used a BEM model with 1,280 nodes (“ico3” in MNE-Python). Three sources were placed randomly in the source space, with the only constraint that they should be at least 3 cm apart. The sources were modeled as current dipoles oriented normal to the local cortical surface. Two scenarios were considered: (a) Type-I: unidirectional interaction between Sources 1 and 2, while Source 3 does not interact with either of the two sources and (b) Type-II: unidirectional interaction between the three sources, with Source 1 driving Source 2, and Source 2 driving Source 3. These two scenarios are depicted in Figure 2.

We used a second-order MVAR model to simulate these scenarios. Brain noise was added by randomly selecting *N*_*b*_ source locations and modeling their activity as uncorrelated pink noise, which was then projected to the sensors and added to the brain signal produced by the three dipoles, as described in the Berlin brain connectivity benchmark (Haufe & Ewald, 2019). In addition to the brain noise, we also added Gaussian measurement noise. Thus, the simulated MEG signal (T = 1,000, J = 1) is given as

$$\begin{array}{l} q_{t}^{s}=\sum_{p=1}^{2}A_{p}q_{t-p}^{s}+v_{t}\\ y_{t}=G\left(r^{s}\right)q_{t}^{s}+{\mathrm{Gq}}_{t}^{b}+e_{t}^{n},\\ \;=y_{t}^{s}+y_{t}^{b}+e_{t}^{n} \end{array}$$ (25)

where **r**^*s*^ ∈ ℝ^*N*_*s*_×3^ are the locations of the *N*_*s*_ = 3 dipoles generating brain signal and $q_{t}^{s}$ are the corresponding amplitudes. The measurement noise is generated according $e_{t}^{n}∼𝓝\left(0,\sigma_{m}^{2}I\right)$.

We fixed SNR_meas_ at 5 and added brain noise for SNR 1, 3, 5, and 10, where brain SNR is defined as

$${\text{SNR}}_{\text{brain}}=\frac{\text{tr}\left\{\sum_{t=1}^{T}\left(y_{t}^{s}\right){\left(y_{t}^{s}\right)}^{\text{T}}\right\}}{\text{tr}\left\{\sum_{t=1}^{T}\left(y_{t}^{b}\right){\left(y_{t}^{b}\right)}^{\text{T}}\right\}}.$$ (26)

Finally, the SNR_meas_ in this scenario becomes

$${\text{SNR}}_{\text{meas}}=\frac{\text{tr}\left\{\sum_{t=1}^{T}\left(y_{t}^{s}+y_{t}^{b}\right){\left(y_{t}^{s}+y_{t}^{b}\right)}^{\text{T}}\right\}}{\text{tr}\left\{\sum_{t=1}^{T}\left(e_{t}^{n}\right){\left(e_{t}^{n}\right)}^{\text{T}}\right\}}.$$ (27)

We generated 50 realizations for each value of SNR_brain_ and each connectivity scenario (noninteracting and interacting sources).

#### Performance evaluation.

We evaluated the performance of our proposed JEDI-MEG method on three criteria: (a) source localization error (SLE), (b) relative error (RE) for the gPDC matrix, and (c) estimation of error in the direction of interaction. We also compared the performance of our proposed method with standard two-step approach of using spatial filtering methods LCMV and MCMV beamformer followed by AR model fitting using ARFIT algorithm.

For the JEDI-MEG approach, the source location is given by the output particle MCMC smoother (see Estimating Source Locations section), which we average over the number of particles and time points. For the LCMV and MCMV approach the source locations are estimated as described in Reference Source Estimation With Beamformers and MVAR Fitting section.

In order to estimate the direction of interaction and consequently compute the true positive rate (TPR) and false positive rate (FPR), we compute the gPDC. We used causal Fourier transform surrogates (100 surrogates, significance level 0.01) to find statistically significant gPDC values (Faes, Porta, & Nollo, 2010).

We define the number of true positives (#TP) as the number of correctly identified links and the number of false negatives (#FN) as the number of missed links. The number of false positives (#FP) is defined as the number of incorrectly estimated links and the number of true negatives (#TN) as the number of correctly identified nonlinks. Thus, TPR and FPR are given as

$$\begin{array}{l} \text{TPR}=\frac{\#\text{TP}}{\#\text{TP}+\#\text{FN}}\\ \text{FPR}=\frac{\#\text{FP}}{\#\text{FP}+\#\text{TN}}. \end{array}$$ (28)

We used bootstrapping technique to obtain the mean and standard deviation (*SD*) for the performance metrics SLE, TPR, FPR, gPDC RE, and *σ*_*m*_ RE as follows: We generated *B* = 100 sets of bootstrap samples of size *S* = 50, obtained by sampling with replacement from the original 50 simulations. In case of SLE, we first computed the average localization error, $\hat{\tau }$^*i*^, over the three sources for each simulation *i* within the bootstrap sample and then computed the mean over the bootstrap sample (SLE_bs_ = $\frac{1}{50}\sum_{i=1}^{50}{\hat{\tau }}^{i}$). Finally, the mean SLE over *B* bootstrap sets was computed as $\frac{1}{100}\sum_{j=1}^{100}{\text{SLE}}_{\text{bs}}^{j}$. In case of other performance metrics, we simply computed the mean value of the metric; first we evaluated each bootstrap sample and then computed the mean of these mean values (obtained for each bootstrap sample) over the *B* bootstrap sets. The *SD* was computed in an analogous manner.

### Simulation Using ECoG Signals

Although MVAR simulations are useful as the ground truth is known, they do not capture the nonlinear characteristics of neural signals. We used electrocorticographic recordings (available for download at https://www.fieldtriptoolbox.org/example/ecog_ny/) as the basis for a realistic simulation of MEG signals for which we still have information on the ground truth. The employed dataset was from a paradigm where the subject was shown images of false fonts, houses, other objects, textures, bodies, text, or faces. For this study, we selected only the trials pertaining to the face stimuli. The ECoG grid had 128 electrodes in total, out of which we selected three electrodes located in the right occipital, right inferior temporal, and right superior temporal sulcus (STS) areas where the electrodes showed largest responses to the face stimuli.

The locations of these three electrodes were mapped on to the individually defined source spaces of the 16 subjects; the locations were in the right lateral occipital parcel (LOC), right fusiform parcel (FG), and right STS parcel of the Desikan–Killiany (DK) atlas.

The ECoG signals were projected to the MEG sensors using the MEG forward model (see Head Modeling section). To account for modeling errors, we used a single-layer BEM model with icosahedron subdivision to 5,120 nodes (using “ico4” in MNE-Python; Gramfort et al., 2014) for data generation and for estimation we used a BEM model with 1,280 nodes (“ico3” in MNE-Python). Measurement and brain noise was added at SNR_meas_ = 3 and SNR_brain_ = 1, respectively, to the simulated MEG signals (see Simulations Based on MVAR Models section). Brain noise was modeled as uncorrelated pink noise generated from randomly selected 2,000 vertices of the source space and projected to the MEG sensors, while the measurement noise was modeled as a Gaussian. Furthermore, to make the simulation scenario more realistic, we set the number of sources, *N*_*s*_, to be estimated by the JEDI-MEG and MCMV algorithms to 5. In reality, we usually do not know the number of sources and thus setting it to a reasonable number is in line with our assumptions (see Notation and Assumptions section). For the MCMV-based source localization, we used the multisource activity index (MAI) as described in Moiseev et al. (2011).

In order to also account for the variability in the temporal structure of the simulated MEG signals, we randomly time-shifted and scaled the amplitudes of the ECoG signal from one subject, so that each of the 16 pseudosubjects have different structure in the simulated signals in addition to the variation introduced by the measurement and biological noise. The amplitudes were randomly scaled between 75% and 125% of the original EcOG data, while the time samples were shifted randomly between −100 and +100 ms using circular shift operation.

### Real MEG Data

We used the data made publicly available by Wakeman and Henson (2015; available at https://legacy.openfmri.org/dataset/ds000117/) and consisting of simultaneous MEG (Elekta Neuromag Vectorview, 204 gradiometers and 102 magnetometers; MEGIN Oy, Espoo, Finland) and EEG recordings (70 channels) from 19 healthy subjects when viewing famous, nonfamous, and scrambled faces. Details on data acquisition and experimental design are in the original publication. Briefly, the subjects were presented with 300 gray-scale photographs; 150 faces of famous people and 150 faces of nonfamous (unknown to the subjects) people. In addition, 150 images of scrambled famous or unknown faces were also presented. Each image was presented twice (either immediately or after five to 15 intervening images). For the sake of simplicity in our analysis, we did not distinguish between the initial and repeated presentations and thus there are three trial types: famous, nonfamous, and scrambled faces. For the purpose of this study, we used only the MEG magnetometer recordings (102 channels) and considered only the trials with nonfamous faces. Signal Space Separation (SSS; Taulu & Simola, 2006) was already applied to the MEG data for the suppression of magnetic interference.

#### Preprocessing.

Due to issues with the data quality, three subjects were excluded (Subjects 1, 5, and 19 in the original dataset). The MEG data of the remaining 16 subjects were preprocessed using MNE-Python (Gramfort et al., 2014), following the guidelines published by Jas et al. (2018). Briefly, the data were band-pass-filtered (zero-phase finite impulse response filter) to 0.3–40 Hz and down-sampled to 220 Hz. Artifacts due to eye blinks and heart beats were removed using independent component analysis, with the maximum number of components to be removed set to 2 for the ocular artifact and 3 for the cardiac artifact. Epochs were extracted −200 … 2,900 ms with respect to the stimulus onset, and the baseline period was also used to estimate the measurement noise covariance using the shrinkage method. Rejection threshold for the trials were set to 4 × 10^−12^ fT for magnetometers and 4 × 10^−10^ fT/cm for gradiometers. We chose 100 trials randomly for the unfamiliar face stimuli and considered the data between 0 and 700 ms for the JEDI-MEG method to estimate the source and connectivity parameters.

#### Head modeling.

We segmented the MRI for the brain volume and for the cortical mantle using the FreeSurfer software (Version 5.1.0; Reuter, Schmansky, Rosas, & Fischl, 2012) with the “recon-all” command. We defined the source space by octahedron subdivision (“oct6” in MNE-Python), resulting in about 4,098 vertices per hemisphere. To compute the MEG forward solution for each subject, we used a single-layer BEM model comprising of just the inner skull surface. We excluded source points that were less than 5 mm to the inner-skull surface. The forward solution was then computed for each source point, assuming the source orientation normal to the local cortical surface.

## RESULTS

### MVAR-Based Simulations

Below, we summarize the results from applying our JEDI-MEG method as well as the reference two-step approaches (MCMV and LCMV beamformers followed by MVAR fitting) to Type-I (unidirectional interaction between two sources and one independent source) and Type-II (unidirectional interaction among three sources) simulation scenarios at several signal-to-noise ratios (SNRs).

#### Source localization.

**Figure 3.**
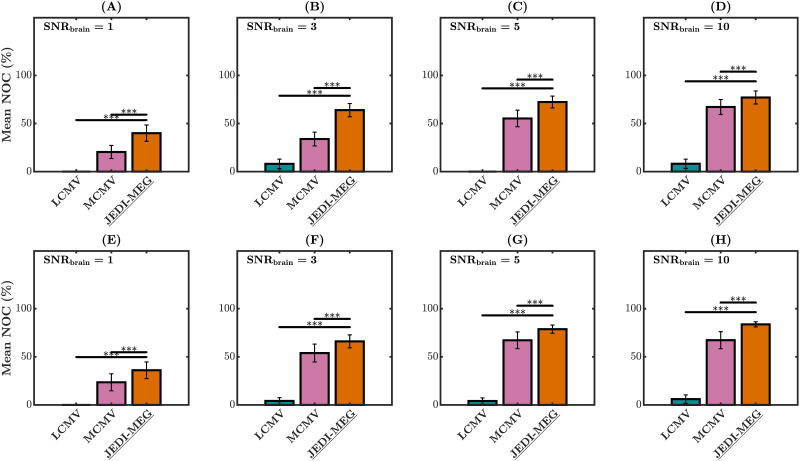
MVAR simulations. The number (percentage) of correctly identified sources (NOC) at SNR_brain_ = 1, 3, 5, and 10 for Type-I (A–D) and Type-II (E–H) scenarios. The measurement noise SNR was fixed to 5, and the error bars represent the standard deviation. *p < 0.05, **p < 0.01, and ***p < 0.001.

Figure 3 shows the mean percentage of cases for which the maximum SLE across the three sources was less than 10 mm, which we refer to as the number of correct hits (NOC). For both Type I (A–D) and Type II (E–H) scenarios, the JEDI-MEG method provided the highest mean NOC compared with the LCMV and MCMV method (*p* < 0.001) at all levels of SNR_brain_. In the case of the LCMV method, the maximum SLE in all three sources remained greater than 10 mm for all simulations in the Type I and Type II scenario for SNR_brain_ = 1 (Figure 3E). For SNR_brain_ = 10, both the JEDI-MEG and MCMV methods provide similar levels of mean NOC for Type-I scenario, while the difference between them remained significant.

**Figure 4.**
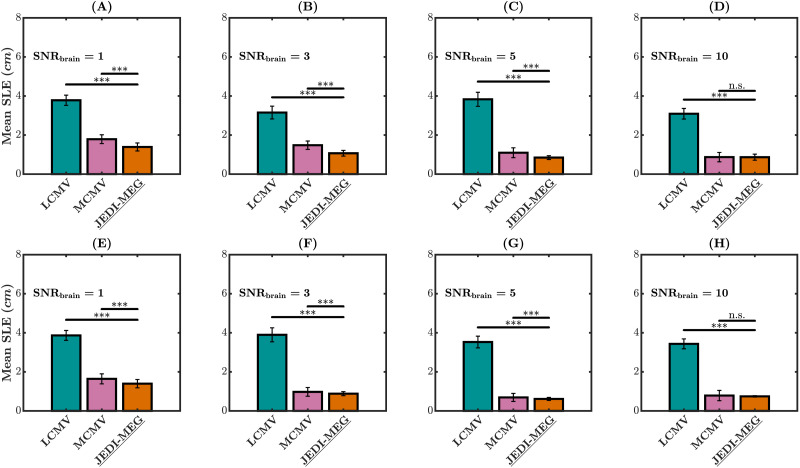
MVAR simulations. SLE at SNR_brain_ = 1, 3, 5, and 10 for Type-I (A–D) and Type-II (E–H) scenarios. The measurement noise SNR was fixed to 5, and the error bars represent the standard deviation. *p < 0.05, **p < 0.01, and ***p < 0.001.

Figure 4 shows the mean SLE for the LCMV, MCMV, and JEDI-MEG method applied to the Type-I and Type-II simulations. The LCMV method gave the highest mean SLE at all simulated levels of SNR_brain_ for both Type-I and Type-II scenarios. Compared with the MCMV method, the JEDI-MEG method provided lower mean SLE for both Type-I and Type-II scenarios at SNR_brain_ = 1, 3, and 5 while at SNR_brain_ = 10 the difference in mean SLE was not statistically significant.

#### Connectivity estimation.

**Figure 5.**
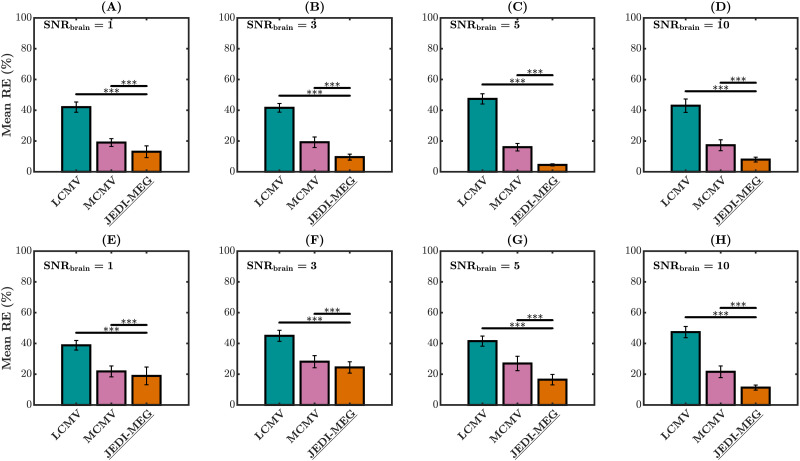
MVAR simulations. REs of connectivity coefficients at SNR_brain_ = 1, 3, 5, and 10 for Type-I (A–D) and Type-II (E–H) scenarios. The measurement noise SNR was fixed to 5, and the error bars represent the standard deviation. *p < 0.05, **p < 0.01, and ***p < 0.001.

Figure 5 shows the mean reconstruction error (RE) for the gPDC matrices obtained using the two-step approach (LCMV and MCMV source estimation followed by AR fitting) and the joint estimation with the JEDI-MEG method. For both Type-I and Type-II simulation scenarios, the JEDI-MEG method gives the lowest RE (*p* < 0.001) compared with both the tested two-step approaches for all values of SNR_brain_.

#### Direction of interaction.

**Figure 6.**
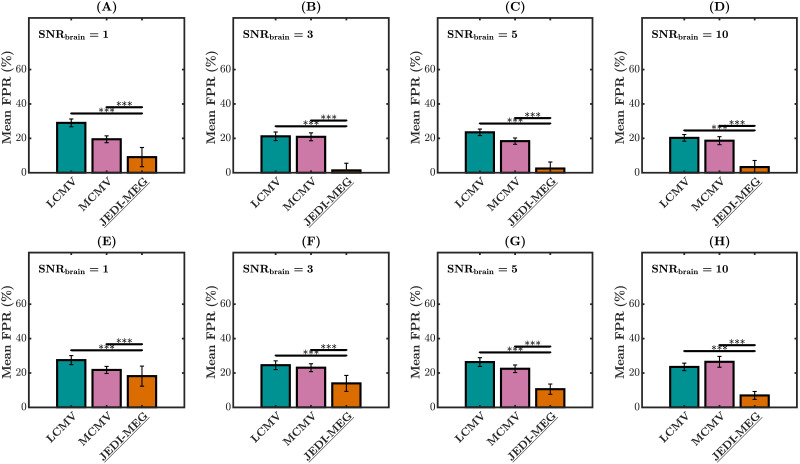
MVAR simulations. FPRs of identified functional connections at SNR_brain_ = 1, 3, 5, and 10 for Type-I (A–D) and Type-II (E–H) scenarios. The measurement noise SNR was fixed to 5, and the error bars represent the standard deviation. *p < 0.05, **p < 0.01, and ***p < 0.001.

The mean FPRs of directed connections identified with the two-step approaches (LCMV and MCMV followed by MVAR-fitting) and with the joint estimation approach using the JEDI-MEG method are shown in Figure 6 for different levels of SNR_brain_. For both the Type-I and Type-II scenarios, the LCMV method followed by AR fitting gave a higher mean FPR compared with JEDI-MEG and MCMV (followed by AR fitting) at SNR_brain=1_. Both the two-step approaches gave comparable mean FPR for SNR_brain>1_. In comparison, the JEDI-MEG method outperformed both LCMV and MCMV (followed by AR-fitting method) in both Type-I and Type-II simulations by yielding lower mean FPR at all noise levels (*p* < 0.001).

**Figure 7.**
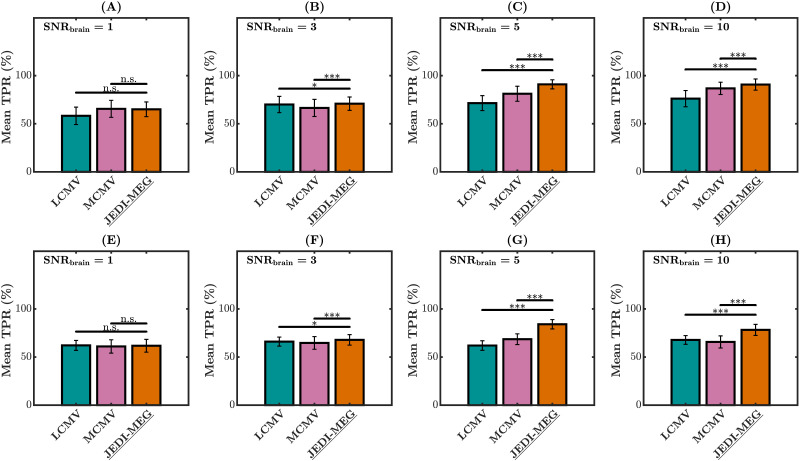
MVAR simulations. TPRs of identified functional connections at SNR_brain_ = 1, 3, 5, and 10 (A–D) for interacting sources. The measurement noise SNR was fixed to 5, and the error bars represent the standard deviation. *p < 0.05, **p < 0.01, and ***p < 0.001.

Figure 7A–D shows the mean TPRs of identified connections for Type-I and Type-II scenarios at different levels of SNR_brain_. For both Type-I and Type-II simulation scenarios, the JEDI-MEG method gave higher mean TPR (*p* < 0.001) compared with two-step approach with LCMV and MCMV method for SNR_brain_ > 3. In cases of SNR_brain_ < 3, all methods gave comparable mean TPR with no statistically significant difference between the JEDI-MEG method and the two-step approaches at SNR_brain_ = 1 both Type-I and Type-II scenarios. Overall, we observe that all the methods gave rather similar mean TPR as SNR_brain_ decreases (although the differences were statistically significant in certain cases) when compared with the substantial differences in the mean FPR results.

### ECoG-Based Simulations

We applied the LCMV- and MCMV-based two-step method and our JEDI-MEG joint estimation method to the MEG data generated based on real ECoG-recorded responses to face stimuli. The data were simulated for 16 subjects.

During analysis, the number of sources was assumed to be 5 for all the methods and the model order was set to 14 based on BIC criterion.

**Figure 8.**
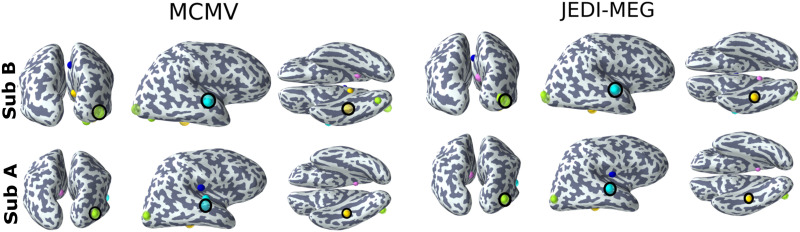
ECoG-based MEG simulations. Source estimates obtained by the MCMV and JEDI-MEG approaches for two example subjects. The estimated (no outline) and true (black outline) sources are shown in green (Source 1), yellow (Source 2), and cyan (Source 3). The estimated extra sources are in blue and magenta.

Figure 8 shows the source estimates obtained with MCMV and JEDI-MEG method in two representative subjects. For Subject A, the MCMV method mislocalizes Sources 1 and 2, while the JEDI-MEG method correctly localizes all three sources. For Subject B, both the MCMV and JEDI-MEG methods achieve comparable localization accuracy.

**Figure 9.**
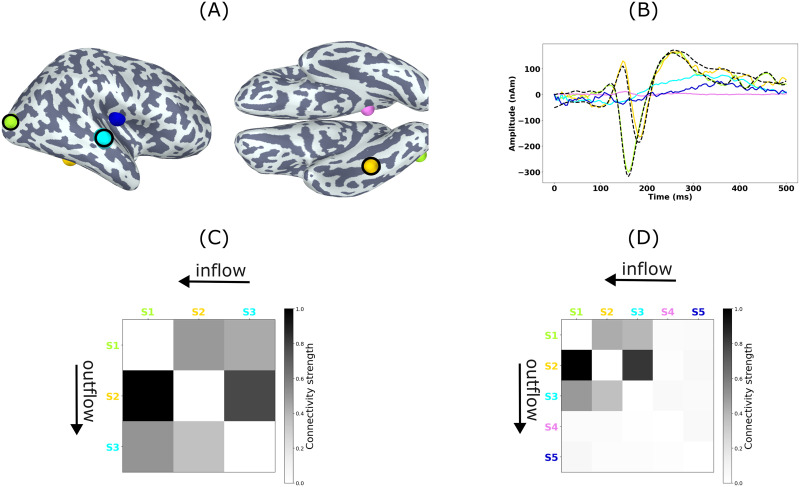
ECoG-based MEG simulations in one subject analyzed with JEDI-MEG. Ground truth and estimates of (A) source locations and (B) source amplitudes. Source 1 (S1, green), Source 2 (S2, yellow), and Source 3 (S3, cyan) with the true locations encircled in black and the true time courses shown as dashed black lines. The extra (inactive) sources are shown in magenta and blue (S4 and S5). (C) The ground-truth and (D) estimated connectivity from MVAR coefficients. The columns and rows represent outward and inward connections.

Figure 9A shows the JEDI-MEG-estimated locations of the three active sources and of the two extra (quiet) sources (we specified the algorithm to find five sources). Figure 9B shows that the estimated temporal dynamics of the three active sources match well with the ground truth, that is, the ECoG measurements. The estimated amplitudes of the two extra sources remain close to zero, also in agreement with the ground truth. Figure 9C shows the ground-truth connectivity matrix, while panel D of that figure shows the corresponding estimate by JEDI-MEG. The connectivity matrix was generated by computing the maximum of the absolute MVAR coefficients across all model orders and subsequently normalizing this matrix by dividing it by its maximum value. Our results reveal bidirectional connectivity between the sources in the right lateral occipital (Source 1) and right fusiform (Source 2) parcel of the DK atlas, with a stronger connection from Source 1 to Source 2. The directed connections from Source 3 to Sources 1 and 2 are also seen. The estimates of the connectivity matrix obtained with JEDI-MEG correspond well with the ground truth shown in Figure 9C.

**Figure 10.**
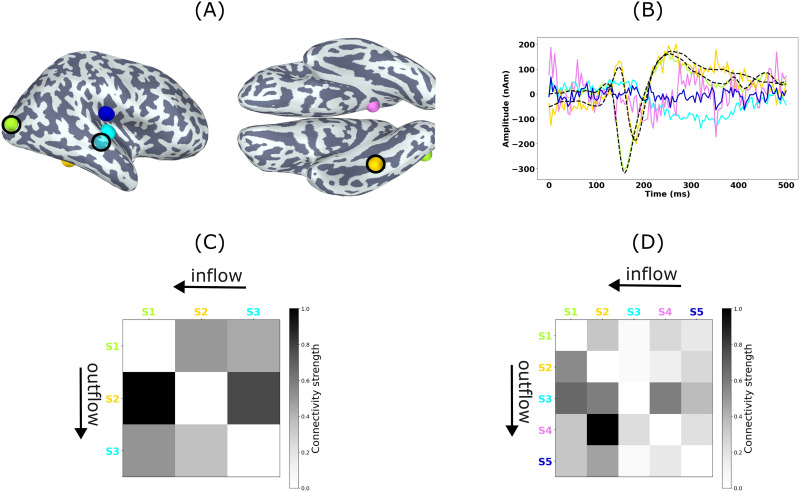
ECoG-based MEG simulations in one subject analyzed with the two-step approach (MCMV beamformer and MVAR fitting). Ground truth and estimates of (A) source locations and (B) source amplitudes. Source 1 (S1, green), Source 2 (S2, yellow), and Source 3 (S3, cyan) with the true locations encircled in black and the true time courses shown as dashed black lines. The extra (inactive) sources are shown in magenta and blue (S4 and S5). (C) The ground-truth and (D) estimated connectivity from MVAR coefficients. The columns and rows represent outward and inward connections.

When applying the MCMV method followed by MVAR fitting for the example case shown in Figure 10, the estimated locations of Sources 1 and 2 are in a good agreement with the ground truth and comparable in accuracy to the locations obtained with the JEDI-MEG approach, while the localization error for Source 3 is clearly larger; see Figure 10A. The estimated amplitudes of Sources 1 and 2 correspond well to the ground truth but the estimated amplitude of Source 3 deviates from the ground truth, particularly in the first 200 ms; see Figure 10B. The estimated orientation, and hence, the amplitude of Source 3 are sign-flipped with respect to the ground truth; yet, the estimate captures the slow response around 300 ms. Overall, ignoring the sign flip, the amplitude of Source 3 as estimated by MCMV deviates from the ground truth more than the estimate by JEDI-MEG. The two extra sources show considerable amplitudes compared with the JEDI-MEG estimates, where the amplitudes were close to zero. The connectivity matrix obtained by MVAR fitting to source amplitudes shows bidirectional connectivity between Source 1 (in the right LOC of the DK atlas) and Source 2 (in the right FG of the DK atlas), with a stronger connection from the superior to inferior source; see Figure 10D. However, there are several spurious connections due to the extra sources that were estimated to be active (Source 2 → Source 4, Source 4 → Source 3). The connections from Source 3 to Sources 1 and 2 are also missing.

**Figure 11.**
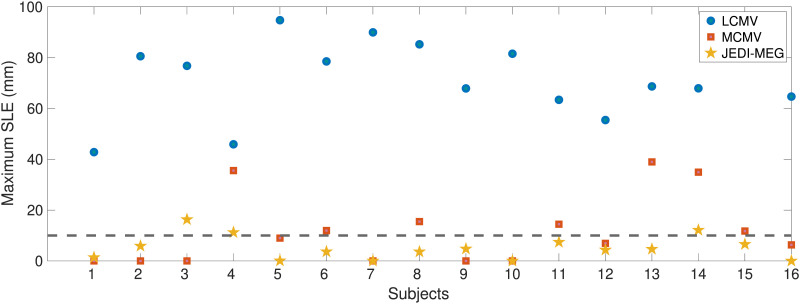
ECoG-based MEG simulations. Maximum SLE across the three sources for the LCMV, MCMV, and JEDI-MEG methods.

After introducing the variability on the temporal structure of the EcOG signal for each subject, we compared the source localization accuracy of the three methods by computing the maximum SLE across the three true sources for each of the 16 subjects, which is shown in Figure 11. The JEDI-MEG method gave a maximum SLE of less than 10 mm in most subjects (13 out of the 16), which was better than what was achieved with the MCMV and LCMV methods. The LCMV had the worst performance, with maximum SLE exceeding 40 mm in all the subjects. The MCMV method yielded a maximum SLE of less than 10 mm in 9 out of the 16 subjects.

**Figure 12.**
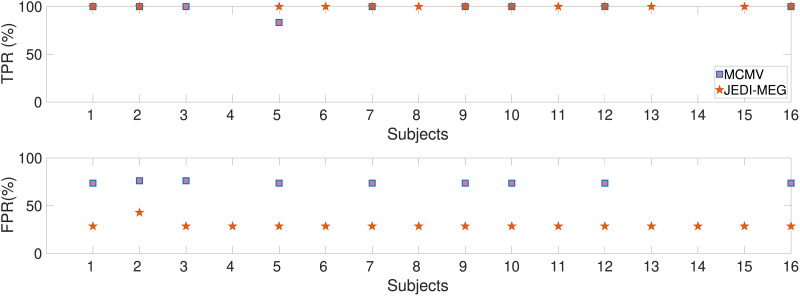
ECoG-based MEG simulations. TPR and FPR for the JEDI-MEG method and the two-step approach using MCMV.

We also computed the connectivity matrices for subjects with maximum SLE < 10 mm. Since the LCMV method did not yield SLE < 10 mm for any of the subjects, Figure 12 shows the TPR and FPR for the two-step approach of MCMV method followed by AR fitting and the proposed joint estimation approach JEDI-MEG. In each case, an AR model (P = 14 as determined by BIC) was fit to the ECoG signal after introducing variability in time and amplitude. The maximum of absolute MVAR coefficients across all the model order was then used as the true connectivity matrix. Connectivity matrices were obtained for JEDI-MEG method and MCMV followed by MVAR fitting in a similar fashion, that is, by obtaining maximum of absolute MVAR coefficients across all the model orders. Finally, in all cases the connectivity matrices were normalized by its (off-diagonal) maximum value. In case of the two-step approach, the MVAR matrix is obtained by fitting an MVAR model to the MCMV estimated source activity, while in case of the JEDI-MEG method, the MVAR matrix is obtained through the ML estimate within the SAEM approach.

To compute the TPR and FPR based on off-diagonal entries, the connectivity matrices were converted to binary matrix using a threshold of 0.05 (i.e., 5% of the maximum value). Since the number of sources was set to 5, while the number of true sources was 3, any connection between the extra source and other sources was counted as a false positive. We can see that the JEDI-MEG approach provides lower FPR compared with the two-step approach with the MCMV method, while the TPR was comparable.

### Real MEG Data

We applied our JEDI-MEG algorithm to real MEG data recorded while the participants were presented with images of human faces (Wakeman & Henson, 2015). Similarly as in the ECoG-based simulations, we set the algorithm to find five sources. The model order was set to 15.

#### Single-subject analysis.

**Figure 13.**
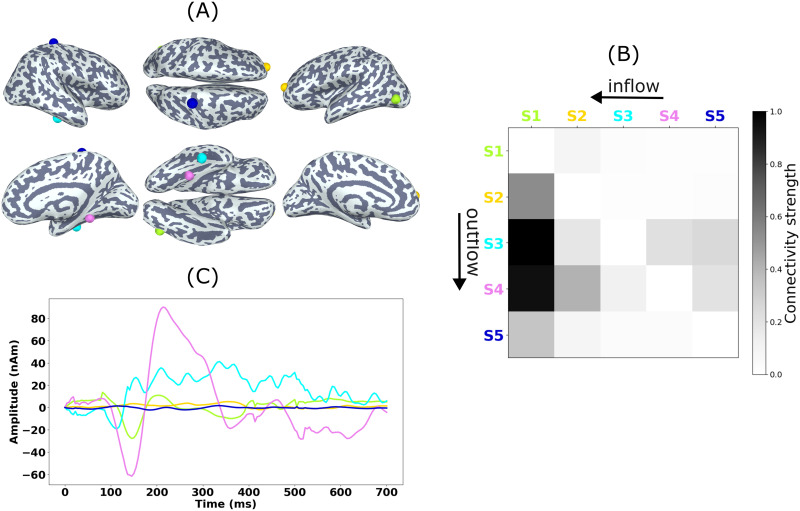
Real MEG data. JEDI-MEG-estimated source locations (A), directed functional connections (B), and amplitudes (C) in one subject. The connectivity matrix represents the maximum absolute coefficients of the MVAR matrix across all model orders (P = 15) and is normalized to the maximum value. In the connectivity matrix, the columns and rows represent outward and inward connections.

Figure 13 shows the estimates of the source dipole locations (averaged over time), amplitudes, and functional connectivity for a representative subject (SUB-07). Active sources were located in the left lateral occipital cortex (Source 1; peak at around 120 ms and 140 ms), right inferior temporal cortex (Source 3; first peak at around 120 ms), and right fusiform gyrus (Source 4; peak at around 150 ms and 210 ms). Sources with relatively lower amplitudes were localized at left orbitofrontal cortex (Source 2) and superior parietal cortex (Source 5). The directed functional connectivity as given by the maximum absolute coefficients of the MVAR matrix across all model orders (P = 14) shows strongest connection from Source 1 (lateral occipital cortex) to Source 3 (inferior temporal cortex) and Source 4 (fusiform gyrus). Bidirectional connections were also observed between Sources 3 (inferior temporal cortex) and 4 (fusiform gyrus) as well as between Source 2 (orbitofrontal cortex) and Source 1 (lateral occipital cortex).

#### Group analysis.

**Figure 14.**
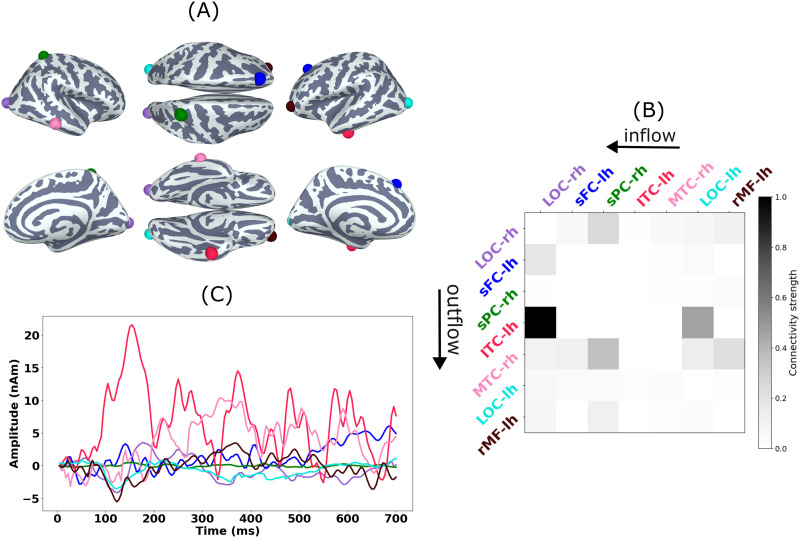
Real MEG data. Group-level (*N* = 16) estimates of source locations (A), directed functional connections (B), and amplitudes (C) by JEDI-MEG. The indicated locations are the source-cluster centroids, the time courses are the averages of those of individual sources in each cluster, and the connectivity plots are derived from the connectivity matrices averaged across subjects. In the connectivity matrix, the columns and rows represent outward and inward connections.

After estimating the source and connectivity parameters individually for all the subjects, we aggregated group-level results using clustering. To this end, we morphed the source locations of each subject to an average brain (“fsaverage” of FreeSurfer). We then grouped the sources using a hierarchical clustering algorithm with a distance threshold. The cluster centroids, given as the spatial mean of the source locations within each cluster, are shown in Figure 14. The centroids are located in the following regions of the DK atlas: left and right lateral occipital cortex, left inferior temporal cortex, right middle temporal cortex, left superior frontal cortex, right superior parietal cortex, and left rostral middle frontal cortex.

Figure 14 also shows the combined amplitude of the sources (using the “flipped-mean” method) within each cluster. Sources in the occipital and inferior temporal cortex again exhibit distinct peaks at 120 and 170 ms. Finally, the directed functional connectivity at the group level was obtained by averaging the subject-specific connectivity matrix pertaining to the sources within each cluster. The strongest connectivity was again observed between the occipital and inferior temporal areas. Although the connection was bidirectional, stronger flow was observed from occipital to ventral parts of the temporal region.

## DISCUSSION

In the present study, we propose a method to jointly estimate the locations and amplitudes of neural sources and their functional connections from MEG data. We use a conditionally linear state-space formulation, where the time-delayed correlation between the sources is given by an MVAR model, whose spectral representation can reveal directed interactions between the sources at specific frequencies. Given the number of sources, our algorithm uses a CPS-AS to estimate the source locations and a Kalman smoother to estimate the source amplitudes. The MVAR matrix and the covariance matrices of process and measurement noise are then obtained as the ML estimates within the SAEM framework. Using MVAR simulations with a simple source structure as well as more realistic ECoG-based simulations of MEG measurements, we show that the proposed joint estimation method—coined JEDI-MEG—outperforms the traditional two-step approach used for connectivity estimation. To further demonstrate the performance of our method, we also applied it to real MEG data containing responses to visually presented faces.

Fukushima and colleagues, driven by a similar motivation as ours, have developed a joint method to estimate functional connectivity using a dynamic variational Bayesian approach (Fukushima et al., 2015). However, their method still requires an anatomical prior in the form of structural connectivity to constrain the MVAR matrix; such prior information may not always be available or even reliable. Dynamical causal modeling (Friston et al., 2003) is another hypothesis-driven approach that requires prior specification of source locations and their interaction structure, which also may not always be known. In contrast to these two approaches, our method only assumes that the MEG data can be explained by a small number of sources (<5), modeled as current dipoles. Our method can then jointly estimate the location, amplitude, and connectivity between these sources without requiring any anatomical constraints or prior specifications of source locations or ROIs.

### Source Estimation

The accuracy of estimating the locations of the sources underlying MEG responses naturally depends on the SNR of the data. Unaveraged MEG responses are typically considered to have an SNR of 3–5; a study on MEG responses to faces estimated an empirical SNR of 4.0–4.3 (Henson, Mouchlianitis, & Friston, 2009). When SNR_brain_ ≥ 3.0 in the MVAR simulations, our JEDI-MEG algorithm recovered more than 80% of the sources with less than 10-mm error (Figure 3). Thus, our algorithm should show at least decent source-localization accuracy already for unaveraged responses, and in trial-based MEG the accuracy shall improve with response averaging, from which the algorithm is designed to benefit.

With the ECoG-based simulations presented in this paper, the JEDI-MEG algorithm estimated sources using an average of 40 trials. Despite the introduced modeling error and low SNR_brain_ = 1, the JEDI-MEG method gave accurate localization results with 13 out of the 16 subjects having a maximum SLE of 10 mm. In contrast, the MCMV-based source localization approach provided SLEs less than 10 mm in only nine out of the 16 subjects. It has been previously shown that MCMV-based algorithms give reliable source estimates even with arbitrary noise covariance matrices (Moiseev et al., 2011). However, in our simulations, we found that the MCMV-approach failed to provide source localization accuracy comparable with that by the JEDI-MEG approach for low SNR_brain_, particularly when analyzing the realistic ECoG-based simulations with modeling errors.

In the JEDI-MEG algorithm, we employ the source estimates provided by the MCMV algorithm to initialize the trajectory of the *N* − *p*th particle. In cases where the MCMV estimates were incorrect, we noticed that the JEDI-MEG approach still converged to the correct locations (Figure 11). This feature is demonstrated also in Figure 11, where the sources in seven subjects were incorrectly localized by the MCMV algorithm but the JEDI-MEG approach nevertheless achieved a low localization error (maximum SLE ≤ 10 mm).

There has recently been interest in exploiting MCMV beamformers for estimating functional connectivity in MEG data (Nunes et al., 2020). Similarly to our JEDI-MEG, the MCMV approach assumes that a few point-like sources (*N*_s_ < 8) are responsible for generating the MEG data (Nunes et al., 2020), and the number of these sources must be specified a priori. If the given number is higher than the true number of sources, the extra sources should ideally be estimated to have close-to-zero amplitude. We found that JEDI-MEG indeed shows baseline-noise-level amplitudes for these extra sources while the MCMV algorithm yielded considerably higher amplitudes, leading to spurious connections between the extra and true sources as seen in Figure 10.

### Connectivity Estimates

We employed simple MVAR-based simulations to compare the three algorithms: MCMV and LCMV followed by MVAR-fitting, and the proposed JEDI-MEG algorithm.

In both Type-I and Type-II scenarios (Figure 2), the LCMV method followed by MVAR fitting yielded more false positives compared with the MCMV and JEDI-MEG approaches. In comparison with the MCMV approach, the JEDI-MEG method clearly outperformed it by giving significantly less false positives even for very low brain noise (SNR_brain_ = 1).

The proposed JEDI-MEG method outperformed both two-step approaches also at identifying true positives, although the differences in the mean TPR were not as substantial (although significant in some scenarios, see Figure 7) as in the FPRs. We can thus conclude that our joint estimation approach consistently gave less false positives compared with the two-step approaches, while providing at least as many true positives.

MCMV beamforming followed by MVAR fitting outperformed the LCMV method at identifying false connections, which was to be expected since the MCMV method has been shown to be insensitive to source correlations (Moiseev et al., 2011).

JEDI-MEG and the SAEM algorithm in it utilizes ML estimation of connectivity parameters, and it is known that ML gives asymptotically unbiased estimates with an increasing sample size. In addition, since we also estimate process-noise covariance explicitly, our approach leads to more accurate estimation of the MVAR matrix. In contrast, as shown in Cheung et al. (2010), the two-step approaches try to fit the MVAR model to the estimated source signals without taking into account the noise, which leads to biased MVAR estimates, particularly at low SNRs. The MCMV approach, despite being less affected by source correlations, yields biased source amplitudes for this reason, which eventually affects connectivity estimation. In two-step MVAR modeling of connectivity, it is common to preselect the locations of the sources or ROIs. Despite not requiring such information, our method gives connectivity estimates which are similar and in many cases superior in accuracy to those obtained with two-step approaches for correlated sources.

### Application to Real Data

We applied JEDI-MEG to real MEG data recorded while the subjects were viewing still images of human faces. JEDI-MEG estimated sources in the occipital, temporal, and frontal cortices, which are the brain regions typically associated with processing of faces (see, e.g., Haist & Anzures, 2017; Haxby, Hoffman, & Gobbini, 2000). In addition, these locations are in agreement with previous MEG/EEG studies that have utilized either dipole localization (Itier & Taylor, 2002; Shibata et al., 2002; Watanabe, Kakigi, Koyama, & Kirino, 1999) or distributed source imaging methods (Meeren, de Gelder, Ahlfors, Hämäläinen, & Hadjikhani, 2013). The sources estimated by JEDI-MEG were also largely consistent across the 16 subjects. The source time courses peaked at around 120 ms and 170 ms, again in agreement with previous studies (Itier & Taylor, 2002; Watanabe et al., 1999).

Although the location and latencies of sources involved in face processing have been extensively studied, it is still not clear how these regions interact. Studies utilizing fMRI have reported correlations between occipital face area (OFA), fusiform face area (FFA), and STS (Davies-Thompson & Andrews, 2012), whereas directed interactions from OFA to FFA and from FFA to STS has been reported (Fairhall & Ishai, 2007). The abovementioned study by Fukushima and colleagues also reported significant interaction from OFA to FFA (Fukushima et al., 2015). A recent study utilizing DCM on MEG data also found reciprocal connections between OFA and FFA (He & Johnson, 2018). Although a rigorous investigation of the existence of these connections and their dynamics is beyond the scope of this paper, we find that our results are largely in agreement with previous results regarding functional connectivity during processing of face stimuli.

Our ECoG-based simulations were derived from real measurements that contained responses to viewing faces; we selected three electrodes that registered the strongest responses. These electrodes are located in the lateral occipital cortex, inferior temporal area (located in or in close proximity of the FFA), and near the superior temporal sulcus (see Figure 9). In line with the studies discussed above, an MVAR model fitted to these three signals revealed bidirectional interaction, with the dominant direction being occipital → inferior temporal as well as bidirectional interaction between the superior temporal sulcus and the occipital and infeior temporal areas, which is in agreement with our JEDI-MEG results on real MEG data from a similar experiment: We observed bidirectional interactions between the sources in the occipital and inferior temporal regions, with a stronger connection from the occipital to inferior temporal sources.

### Limitations

Since source localization in our algorithm is based on the likelihood function, in situations where there are two or more sources with very different strengths, the weaker one is likely mislocalized or discarded. This problem is common to other source localization methods as well and is particularly pronounced when the SNR is low. As seen from our results on MVAR simulations, the localization accuracy of the JEDI-MEG method improves with increasing SNR.

One of the limitations of our approach is that it requires specifying the number of sources, which may not always be known. If the number of sources set is higher than the actual number of sources, the algorithm localizes the extra sources to fit the noise, and the amplitudes of these extra sources will be close to zero, and thus they can be discarded post hoc. In case the given number of sources is less than the actual, the algorithm finds the strongest sources. In any case, we do not recommend setting the number of sources very high to keep the computational cost feasible as the Kalman filter scales cubically with the state dimension, which is *N*_s_*P*, where *N*_*s*_ is the number of sources and *P* is the model order. In order to run the algorithm in a reasonable time, we utilized graphics processing units (GPUs), which reduced the computational time by a factor of 10–15 compared with the CPU-only implementation. For *N*_*s*_ = 5, *N*_*p*_ = 500, *J* = 100, and *P* = 14, the algorithm takes approximately 80 s per SAEM iteration with the NVIDIA V100 Tensor Core GPU and requires 300–500 iterations to converge.

Since we did not exclude event-related transients from the MEG-data, it is likely that the functional connectivity is most likely driven by the evoked activity and not by intrinsic brain activity. Future work could address this limitation by removing the event-related transients for, for example, using approaches mentioned in Schoffelen et al. (2017).

Another shortcoming is that we estimate static connectivity from nonstationary data such as ERPs and, which is suboptimal. We have previously proposed a method to estimate dynamic connectivity from M/EEG data using a joint Kalman filter (Tronarp, Subramaniyam, Särkkä, & Parkkonen, 2018). However, this approach requires the source locations to be specified. Future work could thus combine these approaches and attempt to estimate whole-brain dynamic functional connectivity, possibly applying variational Bayes or ensemble Kalman filters. Finally, although we used only MEG data in this work, it should be straightforward to apply the proposed algorithm to EEG.

## CONCLUSIONS

In this study, we proposed a novel framework based on the SAEM algorithm for joint estimation of source and connectivity parameters of neural sources from MEG data. Using both MVAR- and ECoG-based simulations, we demonstrated that the joint estimation framework provides more accurate reconstruction of functional connectivity compared with the state-of-the-art two-step approaches. Using visual-task-related MEG data from 16 subjects, we showed that our method—coined JEDI-MEG—gives physiologically plausible functional connectivity estimates both at the individual and group level.

## ACKNOWLEDGMENTS

This work was supported by Academy of Finland, grant BRAINTRACK (#289108) to L.P.

## SUPPORTING INFORMATION

All data used in this study are from open-access repositories, and the corresponding links are provided in the text. The implementation of the JEDI-MEG algorithm, the code for generating the simulated data, and a script for preprocessing the MEG data are available at https://github.com/narayanps/jediMEG. Supporting information for this article is available at https://doi.org/10.1162/netn_a_00453.

## AUTHOR CONTRIBUTIONS

Narayan Puthanmadam Subramaniyam: Conceptualization; Data curation; Formal analysis; Methodology; Software; Validation; Visualization; Writing – original draft; Writing – review & editing. Filip Tronarp: Methodology; Validation; Writing – review & editing. Simo Särkkä: Conceptualization; Methodology; Supervision; Writing – review & editing. Lauri Parkkonen: Conceptualization; Funding acquisition; Methodology; Project administration; Resources; Supervision; Validation; Writing – review & editing.

## FUNDING INFORMATION

Lauri Parkkonen, Academy of Finland (https://dx.doi.org/10.13039/501100002341), Award ID: 289108.

## Supplementary Material



## TECHNICAL TERMS

Functional connectivity: Statistical interdependencies between neural time series such as EEG, MEG, or fMRI.
Expectation–maximization: An iterative method to find the local maximum likelihood of parameters in statistical models.
Maximum likelihood: It is the maximum value of the likelihood function.
Likelihood: A function that measures how well a statistical model explains the observed data.
State-space model: A model describing the evolution of hidden states according to a Markov process and how observations are related to the states.
Rao–Blackwellization: A technique for reducing the variance of (Bayesian) estimators using conditional distributions.
Kalman filtering: A recursive algorithm for estimating the hidden state of a linear Gaussian state-space model.
Markov chain Monte-Carlo: A class of algorithms to generate samples from probability distributions using Markov chains.
Particle filtering: A recursive Bayesian estimator for estimating the hidden state of nonlinear state-space models from noisy observations, where noise can be non-Gaussian.
Beamformer: A technique based on spatial filtering for estimating sources from MEG/EEG data.
