# Supplementary material for: Joint estimation of source dynamics and interactions from MEG data
Source: Netw Neurosci. 2025 Jul 17;9(3):842–68. doi: 10.1162/netn_a_00453 (PMC12283153; doi:10.1162/netn_a_00453)
Supplement: Supplementary file 1 [file netn-9-3-842-s001.pdf]

Supplementary material for "Joint estimation of source dynamics and interactions from MEG data"

Narayan Puthanmadam Subramaniyam[1], Filip Tronarp[2], Simo Särkkä[2], Lauri Parkkonen[1],

1 Department of Neuroscience and Biomedical Engineering, Aalto University
2 Department of Electrical Engineering and Automation, Aalto University

*** narayan.subramaniyam@aalto.fi**

1 State-space model

We can re-write a P-th order MVAR model as a first-order model as follows:

$$\underbrace{\begin{bmatrix} q_t^{j,1} \\ \vdots \\ q_t^{j,n} \\ \vdots \\ q_{t-p+1}^{j,1} \\ \vdots \\ q_{t-p+1}^{j,n} \end{bmatrix}}_{\tilde{\mathbf{q}}_t^j} = \underbrace{\begin{bmatrix} a_{11,1} & \cdots & a_{1n,1} & \cdots & a_{11,p} & \cdots & a_{1n,p} \\ \vdots & \vdots & \vdots & \vdots & \vdots & \vdots & \\ a_{n1,1} & \cdots & a_{nn,1} & \cdots & a_{n1,p} & \cdots & a_{nn,p} \\ 1 & 0 & 0 & 0 & 0 & \cdots & 0 \\ 0 & \ddots & 0 & 0 & 0 & \cdots & 0 \\ 0 & 0 & \ddots & 0 & 0 & \cdots & 0 \\ 0 & 0 & 0 & 1 & 0 & \cdots & 0 \end{bmatrix}}_{\tilde{\mathbf{A}}} \underbrace{\begin{bmatrix} q_{t-1}^{j,1} \\ \vdots \\ q_{t-1}^{j,n} \\ \vdots \\ q_{t-p}^{j,1} \\ \vdots \\ q_{t-p}^{j,n} \end{bmatrix}}_{\tilde{\mathbf{q}}_{t-1}^j} + \underbrace{\begin{bmatrix} v_t^{j,1} \\ \vdots \\ v_t^{j,n} \\ \vdots \\ 0 \\ \vdots \\ 0 \end{bmatrix}}_{\tilde{\mathbf{v}}_t^j}. \tag{1}$$

The $np \times np$ covariance matrix, $\tilde{\mathbf{V}}$, of process noise $\tilde{\mathbf{v}}_t^j$ is given as

$$\begin{bmatrix} var(v^{j,1}, v^{j,1}) & \cdots & cov(v^{j,1}, v^{j,N_s}) & 0 & \cdots & 0 \\ \vdots & \vdots & \vdots & \vdots & \vdots & \\ cov(v^{j,N_s}, v^{j,1}) & \cdots & var(v^{j,N_s}, v^{j,N_s}) & 0 & \cdots & 0 \\ 0 & \cdots & 0 & 0 & \cdots & 0 \\ \vdots & \vdots & \vdots & \vdots & \vdots & \\ 0 & \cdots & 0 & 0 & \cdots & 0 \end{bmatrix}. \tag{2}$$

2 Conditional particle filter with ancestor sampling

Before introducing conditional particle filter with ancestor sampling (CPF-AS), we will briefly review sequential Monte-Carlo (SMC) technique and then build on the concept of CPF-AS. In

order to give a brief introduction to the standard SMC technique, also known as particle filter, we will consider a general nonlinear SSM of the form

$$\begin{aligned}\mathbf{x}_t &\sim p_\theta(\mathbf{x}_t|\mathbf{x}_{t-1})\\ \mathbf{y}_t &\sim g_\theta(\mathbf{y}_t|\mathbf{x}_t).\end{aligned} \tag{3}$$

Using a particle filter, a sequence of target distributions $p_{\boldsymbol{\theta}}(\mathbf{x}_{1:t-1}|\mathbf{y}_{1:t-1})$ can be approximated as Lindsten et al. [2014]

$$\widehat{p}_{\boldsymbol{\theta}}(\mathbf{dx}_{1:t-1}|\mathbf{y}_{1:t}) = \sum_{i=1}^{N_p} w_{t-1}^i \delta_{\mathbf{x}_{1:t-1}^i}(\mathbf{dx}_{1:t-1}) \tag{4}$$

where $\{\mathbf{x}_{1:t-1}, w_{t-1}^i\}_{i=1}^{N_p}$ is a weighted particle system, with $\sum_i w_{t-1}^i = 1$. Using sequential importance resampling Särkkä [2013], the particles at time $t-1$ are propagated to time t. This entails sampling the ancestor index with

$$P(a_t^i = m) \propto w_{t-1}^m \tag{5}$$

for $i = \{1 \cdots N_p\}$. A new set of particles at time t are then drawn according to

$$\mathbf{x}_t^i \sim q_{\boldsymbol{\theta}}(\mathbf{x}_t|\mathbf{x}_{t-1}^{a_t^i}, \mathbf{y}_t) \tag{6}$$

where $q_{\boldsymbol{\theta}}(\cdot)$ is some suitable proposal kernel. The particle trajectories can now be extended as $\mathbf{x}_{1:t} := \{\mathbf{x}_{1:t-1}^{a_t^i}, \mathbf{x}_t\}$. The normalized weights w_t^i are given as

$$w_t^i \propto \frac{p_{\boldsymbol{\theta}}(\mathbf{y}_t, \mathbf{x}_t|\mathbf{y}_{1:t-1}, \mathbf{x}_{1:t-1})}{q_{\boldsymbol{\theta}}(\mathbf{x}_t|\mathbf{y}_{1:t}, \mathbf{x}_{1:t-1})}. \tag{7}$$

In the CPF-AS method, we set the N_p-th particle trajectory deterministically, i.e., $\mathbf{x}_{1:T}^{N_p} = \mathbf{x}'_{1:T}$ and the sampling given in Equation 6 is done only for particles $i = 1 \dots N_p - 1$. The ancestor index $a_t^{N_p}$ for the N_p-th particle is sampled according to

$$P(a_t^{N_p} = m) \propto w_{t-1}^m p_{\boldsymbol{\theta}}(\mathbf{x}'_t|\mathbf{x}_{t-1}^m). \tag{8}$$

The CPF-AS is a modification of particle Gibbs (PG) algorithm Andrieu et al. [2009]. Thus, the CPF-AS is similar to a particle filter except that the trajectory of one particle, known as the reference trajectory, is fixed *a priori* Lindsten et al. [2016, 2014].

One could also use a particle smoother in the EM algorithm to estimate $p_\theta(\mathbf{x}_{1:T}|\mathbf{y}_{1:T})$. However, this entails a computational complexity of $O(N_p^2 KT)$, where N_p is the number of particles, K is the number of EM iterations, and T is the number of time samples. Using PMCMC with a particle filter as the kernel reduces the computational complexity to $O(N_p KT)$.

In our model, $\mathbf{x}_{1:T} = \{\mathbf{r}_{1:T}, \tilde{\mathbf{q}}_{1:T}\}$. Due to Rao–Blackwellization, we need to use the CPF-AS only to approximate $p_\theta(\mathbf{r}_{1:T}|\bar{\mathbf{y}}_{1:T})$. We sample the particles, i.e., the source locations at time t, according to the model $\mathbf{r}_t^i \sim \mathcal{N}(\mathbf{m}_{t-1}^i, h^2 \boldsymbol{\Xi}_{t-1})$ with

$$\mathbf{m}_{t-1}^i = a\mathbf{r}_{t-1}^{a_t^i} + (1-a)\bar{\mathbf{r}}_{t-1} \tag{9}$$

where $\bar{\mathbf{r}}_{t-1}$ and $\boldsymbol{\Xi}_{t-1}$ are the mean and variance of the Monte-Carlo approximation to $p_\theta(\mathbf{r}_{t-1}|\bar{\mathbf{y}}_{1:t-1})$. We first set the discount factor $\delta \in (0,1]$ and compute the tuning parameter $a = (3\delta - 1)/2\delta$ and the smoothing parameter $h^2 = 1 - a^2$. In this work, we set $\delta = 0.95$.

Due to the non-Markovian nature of $\mathbf{r}_{1:T}|\bar{\mathbf{y}}_{1:T}$, with $\tilde{\mathbf{q}}_{1:T}$ marginalized out Lindsten et al. [2016], Svensson et al. [2014], the ancestor index does not have the simple expression as given in Equation 8. Instead, for our model, the ancestor index of particle N_p is sampled according to $P(a_t^{N_p} = i) \propto w_{t-1}^i p(\bar{\mathbf{y}}_{t:T}, \mathbf{r}'_{t:T}|\mathbf{r}^i_{1:t-1}, \bar{\mathbf{y}}_{1:t-1})$, where $\bar{\mathbf{y}}_t = \sum_{j=1}^J \mathbf{y}_t^j$. Using the notation in Ref. Lindsten et al. [2016], we define $||\boldsymbol{\mu}_{t|1:t}||^2_{\boldsymbol{\Omega}} \triangleq \boldsymbol{\mu}^T_{t|1:t}\boldsymbol{\Omega}\boldsymbol{\mu}_{t|1:t}$, $\mathbf{P}_{t|1:t} \triangleq \boldsymbol{\Gamma}_{t|1:t}\boldsymbol{\Gamma}^T_{t|1:t}$ and $\mathbf{V} \triangleq \mathbf{F}\mathbf{F}^T$. Furthermore, let us define $\tilde{\mathbf{F}} \in \mathbb{R}^{N_sP\times N_sP}$ as

$$\tilde{\mathbf{F}} = \begin{bmatrix} \mathbf{F} & \mathbf{0} \\ \mathbf{0} & \mathbf{0} \end{bmatrix} \tag{10}$$

and $\tilde{\mathbf{G}} \in \mathbb{R}^{M\times N_sP}$ as

$$\tilde{\mathbf{G}}_t = \begin{bmatrix} \mathbf{G}(\mathbf{r}_t^{N_p}) & \mathbf{0} \end{bmatrix}. \tag{11}$$

Finally, we have

$$p(\bar{\mathbf{y}}_{t:T}, \mathbf{r}'_{t:T}|\mathbf{r}^i_{1:t-1}, \mathbf{y}_{1:t-1}) \propto \boldsymbol{\Delta}^i_{t-1}|\boldsymbol{\Gamma}^i_{t-1}|^{-1/2}\exp(-\frac{1}{2}\boldsymbol{\eta}^i_{t-1}) \tag{12}$$

with

$$\begin{aligned} \boldsymbol{\Delta}^i_t &= p(\mathbf{r}'_t|\mathbf{r}^i_{t-1}) \\ \boldsymbol{\eta}^i_t &= ||\bar{\boldsymbol{\mu}}^i_{t|1:t}||^2_{\boldsymbol{\Omega}_t} - 2\bar{\boldsymbol{\lambda}}_t\bar{\boldsymbol{\mu}}^i_{t|1:t} - ||\bar{\boldsymbol{\Gamma}}^i_{t|1:t}(\bar{\boldsymbol{\lambda}}_t - \boldsymbol{\Omega}_t\bar{\boldsymbol{\mu}}^i_{t|1:t}||^2_{\boldsymbol{\Lambda}_t^{-1}} \\ \boldsymbol{\Lambda}^i_t &= \bar{\boldsymbol{\Gamma}}^{i,T}_{t|1:t}\boldsymbol{\Omega}_t\bar{\boldsymbol{\Gamma}}^i_{t|1:t} + \mathbf{I} \end{aligned} \tag{13}$$

where $\bar{\boldsymbol{\Gamma}}^i_{t|1:t} = \frac{1}{J}\sum_{j=1}^J \boldsymbol{\Gamma}^{i,j}_{t|1:t}$ and $\bar{\boldsymbol{\mu}}^i_{t|1:t}$, $\bar{\boldsymbol{\lambda}}_t$ is defined analogously. For $t = T-1\ldots 1$, we define the following backward statistics:

$$\begin{aligned} \boldsymbol{\Omega}_t &= \tilde{\mathbf{A}}^T(\mathbf{I} - \widehat{\boldsymbol{\Omega}}_{t+1}\tilde{\mathbf{F}}\mathbf{M}^{-1}_{t+1}\tilde{\mathbf{F}}^T)\widehat{\boldsymbol{\Omega}}_{t+1}\tilde{\mathbf{A}}^T \\ \boldsymbol{\lambda}^j_t &= \tilde{\mathbf{A}}^T(\mathbf{I} - \widehat{\boldsymbol{\Omega}}_{t+1}\tilde{\mathbf{F}}\mathbf{M}^{-1}_{t+1}\tilde{\mathbf{F}}^T)\widehat{\boldsymbol{\lambda}}^j_{t+1} \\ \widehat{\boldsymbol{\Omega}}_t &= \boldsymbol{\Omega}_t + \tilde{\mathbf{G}}^T_t\mathbf{E}^{-1}\tilde{\mathbf{G}}_t \\ \widehat{\boldsymbol{\lambda}}^j_t &= \boldsymbol{\lambda}^j_t + \tilde{\mathbf{G}}_t\mathbf{E}^{-1}\mathbf{y}^j_t \\ \mathbf{M}_{t+1} &= \tilde{\mathbf{F}}^T\widehat{\boldsymbol{\Omega}}_{t+1}\tilde{\mathbf{F}} + \mathbf{I} \end{aligned} \tag{14}$$

with $\widehat{\boldsymbol{\Omega}}_T$ and $\widehat{\boldsymbol{\lambda}}_T$ given as

$$\begin{aligned} \widehat{\boldsymbol{\Omega}}_T &= \tilde{\mathbf{G}}^T_T\mathbf{E}^{-1}\tilde{\mathbf{G}}_T \\ \widehat{\boldsymbol{\lambda}}^j_T &= \tilde{\mathbf{G}}_T\mathbf{E}^{-1}\mathbf{y}^j_T. \end{aligned} \tag{15}$$

3 Estimating source amplitudes – Kalman filter

For the i-th particle and j-th trial, the predictive mean and covariance of the Kalman filter are given as

$$\begin{aligned} \boldsymbol{\mu}^{i,j}_{t|1:t-1} &= \tilde{\mathbf{A}}\boldsymbol{\mu}^{i,j}_{t-1|1:t-1} \\ \mathbf{P}^{i,j}_{t|1:t-1} &= \tilde{\mathbf{A}}\mathbf{P}^{i,j}_{t-1|1:t-1}\tilde{\mathbf{A}}^T + \tilde{\mathbf{V}}. \end{aligned} \tag{16}$$

For the i-th particle and j-th trial, the updated mean and covariance of the Kalman filter, $\boldsymbol{\mu}^{i,j}_{t|1:t}$ and $\mathbf{P}^{i,j}_{t|1:t}$, are given as

$$\begin{aligned} \boldsymbol{\mu}^{i,j}_{t|1:t} &= \boldsymbol{\mu}^{i,j}_{t|1:t-1} + \mathbf{K}^{i,j}_t(\mathbf{y}^j_t - \mathbf{G}(\mathbf{r}^i_t)\boldsymbol{\mu}^{i,j}_{t|1:t-1}) \\ \mathbf{P}^{i,j}_{t|1:t} &= \mathbf{P}^{i,j}_{t|1:t-1} - \mathbf{K}^{i,j}_t\mathbf{S}^{i,j}_t(\mathbf{K}^{i,j}_t)^T \\ \mathbf{S}^{i,j}_t &= \mathbf{G}(\mathbf{r}^i_t)\mathbf{P}^{i,j}_{t-1|t-1}\mathbf{G}(\mathbf{r}^i_t)^T + \mathbf{E} \\ \mathbf{K}^{i,j}_t &= \mathbf{P}^{i,j}_{t|t-1}\mathbf{G}(\mathbf{r}^i_t)^T(\mathbf{S}^{i,j}_t)^{-1}. \end{aligned} \tag{17}$$

For the i-th particle and j-th trial, the mean and covariance of the Kalman smoother, $\boldsymbol{\mu}^{i,j}_{t|1:T}$ and $\mathbf{P}^{i,j}_{t|1:T}$ for $t = T-1\ldots 1$, are given as

$$\begin{aligned} \boldsymbol{\mu}^{i,j}_{t|1:T} &= \boldsymbol{\mu}^{i,j}_{t|1:t} + \mathbf{J}^{i,j}_t(\boldsymbol{\mu}^{i,j}_{t|1:T+1} - \boldsymbol{\mu}^{i,j}_{t|1:t-1}) \\ \mathbf{J}^{i,j}_t &= \mathbf{P}^{i,j}_{t|1:t}\tilde{\mathbf{A}}^T(\mathbf{P}^{i,j}_{t|1:t-1})^{-1} \\ \mathbf{P}^{i,j}_{t|1:T} &= \mathbf{P}^{i,j}_{t|1:t} + \mathbf{J}^{i,j}_t(\mathbf{P}^{i,j}_{t|1:T+1} - \mathbf{P}^{i,j}_{t|1:t-1})(\mathbf{J}^{i,j}_t)^T. \end{aligned} \tag{18}$$

Finally, the one-lag covariance smoother is given as

$$\mathbf{P}^{i,j}_{t,t-1|1:T} = \mathbf{P}^{i,j}_{t|1:t}(\mathbf{J}^{i,j}_{t-1})^T + \mathbf{J}^{i,j}_t(\mathbf{P}^{i,j}_{t+1,t|1:T} - \tilde{\mathbf{A}}\mathbf{P}^{i,j}_{t|1:t})(\mathbf{J}^{i,j}_{t-1})^T. \tag{19}$$

4 Maximum-likelihood estimation of parameters

Update for $\tilde{\mathbf{A}}$ At iteration k, to maximize $\hat{Q}_k(\boldsymbol{\theta})$ w.r.t $\tilde{\mathbf{A}}$, we set $\frac{\partial Q_k(\boldsymbol{\theta})}{\partial \tilde{\mathbf{A}}} = 0$. Separating only the terms involving $\tilde{\mathbf{A}}$ in $\hat{Q}_k(\boldsymbol{\theta})$ and using Equations 103, 104 and 118 in Ref. Petersen et al. [2008], we obtain

$$\begin{aligned} \frac{\partial Q_k(\boldsymbol{\theta})}{\partial \tilde{\mathbf{A}}} &= -\frac{1}{2}[-\frac{\partial}{\partial \tilde{\mathbf{A}}}tr(\tilde{\mathbf{V}}^{-1}\boldsymbol{\Psi}\tilde{\mathbf{A}}^T) - \frac{\partial}{\partial \tilde{\mathbf{A}}}tr(\tilde{\mathbf{V}}^{-1}\tilde{\mathbf{A}}\boldsymbol{\Psi}^T) + \frac{\partial}{\partial \tilde{\mathbf{A}}}tr(\tilde{\mathbf{V}}^{-1}\tilde{\mathbf{A}}\boldsymbol{\Sigma}\tilde{\mathbf{A}}^T)] \\ &= \tilde{\mathbf{V}}^{-1}\boldsymbol{\Psi} - \tilde{\mathbf{V}}^{-1}\tilde{\mathbf{A}}\boldsymbol{\Sigma}^{\boldsymbol{T}}. \end{aligned} \tag{20}$$

Since $\boldsymbol{\Sigma}$ is symmetric, by setting the above derivative to zero we obtain $\tilde{\mathbf{A}}_{k+1} = \boldsymbol{\Psi}\boldsymbol{\Sigma}^{-1}$.

Update for $\tilde{\mathbf{V}}$ Again, separating the terms involving only $\tilde{\mathbf{A}}$ in $\hat{Q}_k(\boldsymbol{\theta})$ and using Equations 57 and 124 in Ref. Petersen et al. [2008], we get

$$\begin{aligned}\frac{\partial Q_k(\boldsymbol{\theta})}{\partial \tilde{\mathbf{V}}} &= -\frac{JT}{2}\frac{\partial}{\partial \tilde{\mathbf{V}}}\log|2\pi\tilde{\mathbf{V}}| - \frac{1}{2}[\frac{\partial}{\partial \tilde{\mathbf{V}}}tr(\tilde{\mathbf{V}}^{-1}\boldsymbol{\Phi}) - \frac{\partial}{\partial \tilde{\mathbf{V}}}tr(\tilde{\mathbf{V}}^{-1}\boldsymbol{\Psi}\tilde{\mathbf{A}}^T) \\ &- \frac{\partial}{\partial \tilde{\mathbf{V}}}tr(\tilde{\mathbf{V}}^{-1}\tilde{\mathbf{A}}\boldsymbol{\Psi}^T) + \frac{\partial}{\partial \tilde{\mathbf{V}}}tr(\tilde{\mathbf{V}}^{-1}\tilde{\mathbf{A}}\boldsymbol{\Sigma}\tilde{\mathbf{A}}^T)] \\ &= -\frac{JT}{2}\tilde{\mathbf{V}}^{-1} + \frac{1}{2}\tilde{\mathbf{V}}^{-1}[\boldsymbol{\Phi} - \boldsymbol{\Psi}\tilde{\mathbf{A}}^T - \tilde{\mathbf{A}}\boldsymbol{\Psi}^T + \tilde{\mathbf{A}}\boldsymbol{\Sigma}\tilde{\mathbf{A}}^T]\tilde{\mathbf{V}}^{-1}.\end{aligned} \tag{21}$$

Setting the above derivative to zero, we obtain $\tilde{\mathbf{V}}_{k+1} = \frac{1}{JT}[\boldsymbol{\Phi} - \boldsymbol{\Psi}\tilde{\mathbf{A}}^T - \tilde{\mathbf{A}}\boldsymbol{\Psi}^T + \tilde{\mathbf{A}}\boldsymbol{\Sigma}\tilde{\mathbf{A}}^T]$.

Update for σ_m When we only have measurement noise, separating the terms involving only $\mathbf{E} = \sigma_m^2\mathbf{I}$ in $\hat{Q}_k(\boldsymbol{\theta})$, we obtain

$$\begin{aligned}\frac{\partial Q_k(\boldsymbol{\theta})}{\partial \sigma_m} &= -\frac{JT}{2}\frac{\partial}{\partial \sigma_m}\log|2\pi\sigma_m^2\mathbf{I}| - \frac{1}{2}[\frac{\partial}{\partial \sigma_m}tr(\sigma_m^{-2}[\mathbf{Z} - \boldsymbol{\Upsilon}\bar{\mathbf{G}}^T - \bar{\mathbf{G}}\boldsymbol{\Upsilon}^T + \bar{\mathbf{G}}\boldsymbol{\Phi}\bar{\mathbf{G}}^T]) \\ &= -JTM\frac{1}{\sigma_m} + \sigma_m^3 tr(\mathbf{Z} - \boldsymbol{\Upsilon}\bar{\mathbf{G}}^T - \bar{\mathbf{G}}\boldsymbol{\Upsilon}^T + \bar{\mathbf{G}}\boldsymbol{\Phi}\bar{\mathbf{G}}^T).\end{aligned} \tag{22}$$

Again, setting the above derivative to zero we get $\sigma_{m,k+1}^2 = \frac{1}{JMT}tr(\mathbf{Z} - \boldsymbol{\Upsilon}\bar{\mathbf{G}}^T - \bar{\mathbf{G}}\boldsymbol{\Upsilon}^T + \bar{\mathbf{G}}\boldsymbol{\Phi}\bar{\mathbf{G}}^T)$, where M is the number of MEG sensors.

Update for σ_b and σ_m When biological noise is also included, the total noise covariance $\mathbf{R} = \sigma_m^2\mathbf{I} + \sigma_b^2\mathbf{G}\mathbf{G}^T$. Separating terms involving only σ_b^2 and σ_m^2, the update of σ_b and σ_m can be done using gradient descent with backtracking line search Boyd and Vandenberghe [2004], where the goal is to minimize the objective function

$$\mathcal{L}_k(\boldsymbol{\theta}) = -Q_k(\boldsymbol{\theta}) = \frac{JT}{2}\log|2\pi\mathbf{R}| + \frac{1}{2}tr\{\mathbf{R}^{-1}[\mathbf{Z} - \boldsymbol{\Upsilon}\bar{\mathbf{G}}^T - \bar{\mathbf{G}}\boldsymbol{\Upsilon}^T + \bar{\mathbf{G}}\boldsymbol{\Phi}\bar{\mathbf{G}}^T]\} \tag{23}$$

with the following gradients obtained using the chain rule: Petersen et al. [2008]

$$\begin{aligned}\frac{\partial \mathcal{L}_k(\boldsymbol{\theta})}{\partial \sigma_b} &= tr\{(\frac{\partial \mathcal{L}_k(\boldsymbol{\theta})}{\partial \mathbf{R}})^T\frac{\partial \mathbf{R}}{\partial \sigma_m}\} \\ &= tr\{(\mathbf{R}^{-1} - \mathbf{R}^{-1}[\mathbf{Z} - \boldsymbol{\Upsilon}\bar{\mathbf{G}}^T - \bar{\mathbf{G}}\boldsymbol{\Upsilon}^T + \bar{\mathbf{G}}\boldsymbol{\Phi}\bar{\mathbf{G}}^T]\mathbf{R}^{-1})^T(2\sigma_b\mathbf{G}\mathbf{G}^T)\}\end{aligned} \tag{24}$$

and

$$\begin{aligned}\frac{\partial \mathcal{L}_k(\boldsymbol{\theta}))}{\partial \sigma_m} &= tr\{(\frac{\partial \mathcal{L}_k(\boldsymbol{\theta})}{\partial \mathbf{R}})^T\frac{\partial \mathbf{R}}{\partial \sigma_b}\} \\ &= tr\{(\mathbf{R}^{-1} - \mathbf{R}^{-1}[\mathbf{Z} - \boldsymbol{\Upsilon}\bar{\mathbf{G}}^T - \bar{\mathbf{G}}\boldsymbol{\Upsilon}^T + \bar{\mathbf{G}}\boldsymbol{\Phi}\bar{\mathbf{G}}^T]\mathbf{R}^{-1})^T(2\sigma_m\mathbf{I})\}.\end{aligned} \tag{25}$$

5 Source and connectivity estimation with beamformer

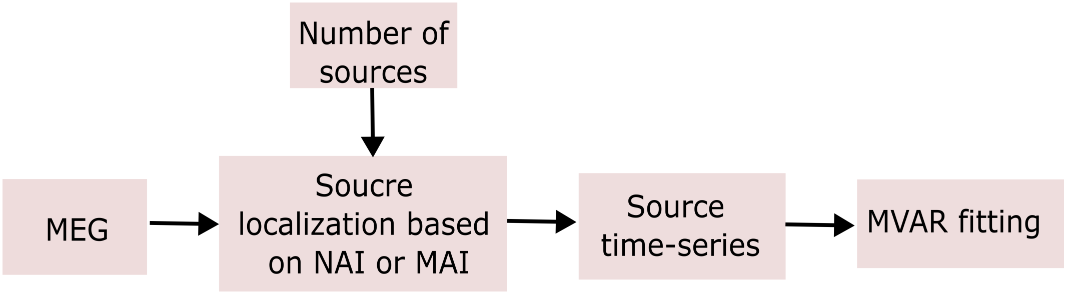

Figure 1: Fixed number of source locations are selected based on highest NAI or MAI values. Source time-series at these locations are estimated using the spatial filter. Once source activity is estimated, we use ARFIT algorithm to estimate the MVAR matrix.

6 JEDI-MEG algorithm

The proposed joint estimation method is summarized in Algorithm 1 and the stochastic E-step used within the joint estimation scheme is presented in Algorithm 2.

Algorithm 1 Joint estimation.

procedure SAEM
    Initialize $\boldsymbol{\theta}_0$ and $\mathbf{r}'_{1:T}[0]$ and $\hat{Q}_0(\theta) = 0$
    for $k \geq 1$ to K **do**
        Run RB-PMCMC to obtain $\{\mathbf{r}^i_{1:T}, w^i_T\}_{i=1}^N$, $\{\boldsymbol{\mu}^{i,j}_{1:T}\}_{i=1,j=1}^{i=N,j=J}$ and $\mathbf{r}_{1:T}[k]$.
        Compute $\hat{Q}_k(\theta)$ in the stochastic E-step.
        Compute $\hat{\boldsymbol{\theta}}_k = \arg\max_\theta \hat{Q}_k(\theta)$ using Equation **??**.
    end for
end procedure

Algorithm 2 Stochastic E-step.

procedure RB-PMCMC
  Input: $\mathbf{y}_{1:T}$, $\bar{\mathbf{y}}_{1:T}$, $\mathbf{r}'_{1:T}[k]$
  Output: $\{\mathbf{r}^i_{1:T}, w^i_T\}_{i=1}^{N_p}$, $\{\boldsymbol{\mu}^{i,j}_{1:T}\}_{i=1,j=1}^{i=N_p,j=J}$ and $\mathbf{r}_{1:T}[k+1]$,
  $\mathbf{r}^i_t \sim p(\mathbf{r}_t)$ for $i = 1 \cdots N_p - 1$ and $t = 1$
  Compute $\boldsymbol{\Omega}_t$ and $\boldsymbol{\lambda}_t$ for $t = 1 \cdots T$ using Equation 14
  Set $\{\mathbf{r}^{N_p}_1 \cdots \mathbf{r}^{N_p}_T\} = \{\mathbf{r}'_1 \cdots \mathbf{r}'_T\}$
  Compute $w^i_t \propto p_{\boldsymbol{\theta}}(\bar{\mathbf{y}}_t | \mathbf{r}^i_t)$ for $i = 1 \cdots N_p$ and $t = 1$
  Compute $\boldsymbol{\mu}^{i,j}_{t|1:t}$ and $\boldsymbol{P}^{i,j}_{t|1:t}$ for $i = 1 \cdots N_p$, $j = 1 \cdots J$ and $t = 1$ Equation 17
  for $t = 2$ to T **do**
    Draw a^i_t with $\mathbb{P}(a^i_t = m) = w^m_{t-1}$ for $i = 1 \cdots N_p - 1$.
    $\mathbf{r}^i_t \sim p_{\boldsymbol{\theta}}(\mathbf{r}_t | \mathbf{r}^{a^i_t}_{t-1})$ for $i = 1 \cdots N_p - 1$.
    Compute $\boldsymbol{\Delta}^i_{t-1}$, $\boldsymbol{\eta}^i_{t-1}$, $\boldsymbol{\Lambda}^i_{t-1}$ for $i = 1 \cdots N_p$ using Equation 13.
    Draw $a^{N_p}_t$ according to $\mathbb{P}(a^{N_p}_t = i) \propto w^i_{t-1} \boldsymbol{\Delta}^i_{t-1} |\boldsymbol{\Lambda}^i_{t-1}| \exp\left(-\frac{1}{2}\boldsymbol{\eta}^i_{t-1}\right)$.
    Set $\mathbf{r}^i_{1:t} = \{\mathbf{r}^{a^i_t}_{1:t-1}, \mathbf{r}^i_t\}$ for $i = 1 \cdots N_p$.
    Set $\boldsymbol{\mu}^{i,j}_{1:t-1|1:t-1} = \boldsymbol{\mu}^{a^i_t,j}_{1:t-1|1:t-1}$ and $\boldsymbol{P}^{i,j}_{1:t-1|1:t-1} = \boldsymbol{P}^{a^i_t,j}_{1:t-1|1:t-1}$ for $i = 1 \cdots N_p$, $i = 1 \cdots N_p$ and $j = 1 \cdots J$.
    Set $\boldsymbol{\mu}^{i,j}_{1:t-1|1:t-2} = \boldsymbol{\mu}^{a^i_t,j}_{1:t-1|1:t-2}$ and $\boldsymbol{P}^{i,j}_{1:t-1|1:t-2} = \boldsymbol{P}^{a^i_t,j}_{1:t-1|1:t-2}$ for $i = 1 \cdots N_p$ and $j = 1 \cdots J$.
    Compute filtered mean $\boldsymbol{\mu}^{i,j}_{t|1:t}$ and covariance $\boldsymbol{P}^{i,j}_{t|1:t}$ for $i = 1 \cdots N_p$ and $j = 1 \cdots J$ using Equation 17.
    Compute predictive mean $\boldsymbol{\mu}^{i,j}_{t|1:t-1}$ and covariance $\boldsymbol{P}^{i,j}_{t|1:t-1}$ for $i = 1 \cdots N_p$ and $j = 1 \cdots J$ using Equation 16.
    $w^i_t \propto p_{\boldsymbol{\theta}}(\bar{\mathbf{y}}_t | \mathbf{r}^i_t, \bar{\mathbf{y}}_{1:t-1})$ for $i = 1 \cdots N_p - 1$.
    Normalize w^i_t s.t. $\sum_i w^i_t = 1$.
  end for
  for $t = T$ to 1 **do**
    Compute smoothed mean $\boldsymbol{\mu}^{i,j}_{t|1:T}$ and covariance $\boldsymbol{P}^{i,j}_{t|1:T}$ for $i = 1 \cdots N_p$ and $j = 1 \cdots J$ using Equation 18.
  end for
  Set $\mathbf{r}[k+1] = \mathbf{r}^M_{1:T}$ with $\mathbb{P}(M = m) = w^m_T$.
end procedure

References

Christophe Andrieu, Arnaud Doucet, and Roman Holenstein. Particle markov chain monte carlo methods. *Journal of the Royal Statistical Society: Series B (Statistical Methodology)*, 72(3): 269–342, 2009.

Stephen Boyd and Lieven Vandenberghe. *Convex optimization*. Cambridge university press, 2004.

F. Lindsten, P. Bunch, S. Särkkä, T. B. Schön, and S. J. Godsill. Rao-blackwellized particle smoothers for conditionally linear gaussian models. *IEEE Journal of Selected Topics in Signal Processing*, 10(2):353–365, 2016.

Fredrik Lindsten, Michael I. Jordan, and Thomas B. Schön. Particle gibbs with ancestor sampling. *Journal of Machine Learning Research*, 15:2145–2184, 2014.

Kaare Brandt Petersen, Michael Syskind Pedersen, et al. The matrix cookbook. *Technical University of Denmark*, 7(15):510, 2008.

Simo Särkkä. *Bayesian filtering and smoothing*, volume 3. Cambridge University Press, 2013.

A. Svensson, T. B. Schön, and F. Lindsten. Identification of jump markov linear models using particle filters. In *53rd IEEE Conference on Decision and Control*, pages 6504–6509, 2014.
